# Transcriptome profiling of *Gerbera hybrida* reveals that stem bending is caused by water stress and regulation of abscisic acid

**DOI:** 10.1186/s12864-019-5961-1

**Published:** 2019-07-22

**Authors:** Yafei Ge, Qixian Lai, Ping Luo, Xiaojing Liu, Wen Chen

**Affiliations:** 1 The Key Laboratory for Quality Improvement of Agricultural Products of Zhejiang Province, School of Agriculture and Food Science, Zhejiang Agriculture & Forestry University, Lin’an, China; 2 The Key Laboratory of Creative Agriculture, Ministry of Agriculture and Rural Affairs, Rural Development Institute, Zhejiang Academy of Agricultural Sciences, Hangzhou, China; 3 Institute of Botany, Jiangsu Province and Chinese Academy of Sciences, Nanjing, China

**Keywords:** *Gerbera hybrida*, Stem bending, Transcriptome, Water stress, Abscisic acid

## Abstract

**Background:**

*Gerbera hybrida* is one of the most popular cut flowers in the world; however, stem bending, which always happens when gerbera flower harvested from the field, greatly limits its vase life. To date the molecular mechanisms underlying stem bending remain poorly understood.

**Results:**

In this study, we performed high-throughput transcriptome sequencing of gerbera during stem bending using the Illumina sequencing technology. Three cDNA libraries constructed from mRNAs of gerbera stem at stem bending stage 0, 2 and 4 were sequenced. More than 300 million high-quality reads were generated and assembled into 96,492 unigenes. Among them, 34,166 unigenes were functionally annotated based on similarity search with known protein. Sequences derived from plants at different stem bending stages were mapped to the assembled transcriptome, and 9,406 differentially expressed genes (DEGs) were identified. Through Kyoto Encyclopedia of Genes and Genomes (KEGG) enrichment analysis, specific pathways were identified during the stem bending process, such as phenylpropanoid biosynthesis pathway, phenylalanine metabolism pathway, starch and sucrose metabolism pathway, and plant hormone signal transduction pathway. A total of 211 transcription factors (TFs), including TF families involved in plant senescence, such as NAC, MYB, WRKY, and AP2/ERF members, as well as TFs related to water stress tolerance, were shown to be regulated during stem bending. Gene Onotology (GO) functional enrichment analysis indicated that key genes involved in responses to osmotic and oxidative stresses were also varied in expression during this process. Furthermore, analysis of DEGs involved in the hormone signaling pathways and determination of endogenous abscisic acid (ABA) content showed that stem bending may be an ethylene-independent process, but regulated by ABA. In short, our findings suggested that the stem bending of cut gerbera may be caused by the involvement of water stress and regulation of ABA during the postharvest life.

**Conclusions:**

The transcriptome sequences provide a valuable resource in revealing the molecular mechanism underlying stem bending of cut flower and offer novel genes that can be used to guide future studies for ornamental plant breeding.

**Electronic supplementary material:**

The online version of this article (10.1186/s12864-019-5961-1) contains supplementary material, which is available to authorized users.

## Background

Gerbera (*Gerbera hybrida*) is one of the most important cut flowers in markets worldwide. Currently the vase life of many cultivars of gerbera flower is short due to the occurrence of stem bending, which always precedes wilting of the ray petals. Stem bending is one of the leading limitations to vase life of gerbera [1]. It is also found in other species of cut flowers, such as chrysanthemum and rose [2, 3], leading to quality loss in the marketing of cut flowers. Therefore how to prevent its occurrence has been a constant focus of flower breeders.

Stem bending has been proved to occur as a result of a complex set of physiological and cellular factors. Previous research suggested that lack of mechanical strength of stem causes the stem bending in cut flowers [1–3]. A key component of mechanical strength is wall thickening, particularly in the xylem [4–6]. Xylem usually consists of water-conducting vessels and tracheids as well as xylem fibres. The other component is the extent of sclerenchyma formation in the stem [1, 7]. Lack of sclerenchyma in the upper parts of the flowering stem tends to hasten stem bending. Both xylem and sclerenchyma cells contain high levels of lignin, the major structural support material, in their secondary walls. The importance of lignin for stem rigidity was experimentally proved in many plants. Earlier study in rose has proved that lignification level of peduncle xylem tissues was positively associated with the tenacity of stem [8]. Higher S/G ratio (S: syringyl-like lignin structures; G: guaiacyl-like lignin structures) was observed in the stronger rose peduncles compared to the weaker ones [2]. In gerbera, similar results were presented and the stems bending earlier had lower lignin concentrations than those not bending [1]. In spray chrysanthemum, *CgCOMT*, that encodes caffeic acid 3-O-methyltransferase and participates in lignin biosynthesis pathway, was expressed prominently in the stem. Expression of *CgCOMT* increased with the development of the pedicel, and was higher in pedicels with greater rigidity [3]. Silencing of genes encoding cinnamyl-alcohol dehydrogenase, an enzyme which is involved in lignin synthesis and mainly expresses in sclerenchyma cells, showed weak mechanical strength of stem in rice, suggesting that lignin deposition in sclerenchyma is important for mechanical strength [9].

Adverse water relation is another main cause of stem bending [1]. Plant cells require sufficient water inflow to keep cell turgor above yield threshold and then the turgor pressure makes a significant contribution to organ mechanical properties. Insufficient turgor pressure disenables stem to support the flower head under its gravity, which accelerates the occurrence of bending. Cut flower may suffer from the adverse water relation due to several environmental factors. The first is water deficit stress, which often happens during postharvest handling and results in abnormal flower opening, wilting of the pedicel and flower [10, 11]. Recently, genome expression profiling under dehydration stress has been analyzed in many plant species, such as rice, barley, and soybean [12–14]. However, little is known about it in cut flower. As one of the few examples, expression profiling of rose flower in response to dehydration was obtained through suppression subtractive hybridization [15]. Fifty-four genes whose expression were positively regulated by dehydration were identified, including genes associated with drought stress (e.g., dehydrin, Suc synthase), transcription factors (e.g., NAC protein, zinc ion-binding protein), and cell wall related genes. The second is net water loss from stem especially in the bending segments. It has been reported that the water balance, calculated as the difference between water uptake and transpiration, was negative during almost the entire vase life. The largest net water loss was found in the segment where bending was mostly located [1]. The third is low water uptake because of xylem blockage by bacteria or material from dead stem cells [16, 17]. Inhibiting bacterial growth in the vase water can reduce the occurrence of stem bending [16]. Despite significant progress during the past decade in aiming to understand the cause of stem bending at anatomical and physiological level in cut flower, the molecular mechanisms that control this process remain obscure.

*G. hybrida* (2n = 2x = 50) belongs to the plant family Asteraceae. Cultivated gerbera probably originates from a crossing of two wild species from Africa and is highly heterozygous (*G. jamesonii* and *G. viridifolia*) [18]. Because of the complicated genetic background, few genomic and genetics resources are currently available for gerbera, which limits the gerbera breeding and biology research. The generation of Illumina-based RNA sequencing (RNA-Seq) technology provides a unique opportunity for creating transcriptomic data for non-model plant species, particularly those without reference genome sequence data and those with high levels of heterozygosity [19]. To date, transcriptome analysis of gerbera has been conducted and most researches focused on identification of gene families related to organ growth and development because of its typical complex inflorescence, including flower organ differentiation [20], petal organogenesis [21], petal growth [22, 23] and disease resistance [24]. However, such database has not provided a global overview of the molecular mechanisms underlying the stem bending in gerbera.

In this study, we performed large-scale transcriptome sequencing of gerbera plants during different bending stages using the Illumina sequencing technology. Differentially expressed genes (DEGs), enrichment metabolic pathways and biological process in the stem bending process were analyzed. Further detailed analysis of key DEGs provided some novel insights into stem bending of gerbera and offered candidate genes or markers which can be used to guide future efforts attempting to breed stem bending resistant gerbera.

## Methods

### Plant material and sampling

Gerbera (*G. hybrida*) ‘Monetta’, a popular cut-flower type cultivar with lilac floret and black pappus, was used in this study. Gerbera plants were obtained from greenhouse in Zhejiang agriculture & forestry university. Flowers were harvested when mature stamens appeared in two outer whorls of flowers in the floral head [1]. After harvest by hand, flowers were immediately placed in water and delivered to the laboratory within 3 h. Upon arrival, uniform flowers were selected with a similar stem. Then, their stems were cut to a length of 40 cm under water, and the flowers were held in deionized water at 20 °C and 60% RH. Flowers were individually placed in 20 cm high plastic bottles containing 250 mL demineralized water, and the stems were kept at an angle of 20° with respect to the vertical. Stem bending stage was defined as described by Perik (2012) with minor modifications as follow: stage 0, the angle between floral head surface and horizontal line is less than 30°; stage 1, the angle is between 30° and 60°; stage 2, the angle is between 60° and 90°; stage 3, the angle is between 90° and 120°; stage 4, the angle is between120° and 150°; stage 5, the angle is between 150° and 180° (Additional file 1: Figure S1) [1]. The stem bending stage was determined daily. The stem segment (7–12 cm below the floral head) where bending usually occurs in ‘Monetta’ flower in our previous work were collected from flower at bending stage 0, 2 and 4, respectively. For the high-throughput sequencing, two biological replicates were collected from stem pools of six cut flowers at each bending stage and immediately frozen in liquid nitrogen.

### Total RNA extraction, RNA-seq library construction and sequencing

TRIzol® reagent (Invitrogen, USA) was used for total RNA extraction according to the manufacturer’s instructions. The quality and quantity of total RNA was measured by NanoDrop 2000c UV-Vis Spectrophotometer (Thermo Fisher Scientific Inc.) and the quality of RNA samples was assessed by agarose gel electrophoresis.

At each bending stage, the RNA samples from the six individuals were mixed in equal amounts to generate one sample. These mixed RNA samples were subsequently used for cDNA library construction and Illumina deep sequencing by Majorbio Biotech Co., Ltd., Shanghai, China. Briefly, 5 μg of total RNA in each group was used to construct libraries by using a TruSeq™ RNA sample preparation Kit (Illumina, USA). Ribosomal and viral RNA were removed and Poly (A) + mRNA was isolated with oligo (dT) beads (Invitrogen, USA). Then the mRNA was randomly fragmented using fragmentation buffer to perform cDNA synthesis, end repair, A-base addition and ligation of the Illumina-indexed adaptors according to the Illumina protocol. Libraries were size-selected on 2% Low Range Ultra Agarose for cDNA target fragments of 300–500 bp, followed by PCR amplification using Phusion DNA polymerase (NEB) for 15 PCR cycles. Amplicons were collected and purified by Certified Low Range Ultra Agarose (Bio-Rad, USA) gel electrophoresis. Following quantification by TBS-380 micro fluorometer with Picogreen® reagent (Invitrogen, USA), clone clusters were generated on Illumina cBot, using Truseq PE Cluster Kit v3-cBot-HS. Then, high-throughput sequencing was performed on an Illumina Miseq sequencer, using Truseq SBS Kit v3-HS 200 cycles.

### RNA-seq data processing, de novo assembly

The raw paired end (PE) reads were cleaned by removing adapter sequences, empty reads, and low-quality reads (reads with over 10% unknown base pairs ‘N’) using SeqPrep and Sickle software [25, 26]. Due to the absence of a reference genome, three libraries from different bending stages were utilized to perform de novo assembly of the gerbera transcriptome, using Trinity de novo transcriptome assembly software [27].

### Sequence annotation and classification

For annotation, the sequences were searched against the NCBI non-redundant (NR) protein database [28] using BlastX, with a cut-off E-value of 10^− 5^. Gene ontology (GO) terms were extracted from the annotation of high-score BLAST matches in the NCBI NR proteins database (E value ≤1.0 × 10^− 5^) using blast2go [29], and then sorted for the GO categories using in-house perl scripts [30]. Functional annotation of the proteome was carried out by a BlastP homology search against the NCBI Clusters of Orthologous Groups (COG) database [31]. Kyoto Encyclopedia of Genes and Genomes (KEGG) pathway annotations were performed using Blastall software against the KEGG database [32]. TFs were identified and classified into different families using Plant Transcription Factor Database and iTAK pipeline [33–35].

### Gene expression analysis

After assembling the transcriptome, every RNA-seq library was separately aligned to the generated transcriptome assembly, using Bowtie [36]. The counting of alignments was performed using the RSEM package [37]. The DEGs were analyzed using the R Bioconductor package, edgeR [38]. The *P*-value set the threshold for the differential gene expression test. The threshold of the P-value in multiple tests was determined by the value for the false discovery rate (FDR) [39]. We used ‘FDR ≤ 0.05 and the absolute value of log_2_ fold change (log_2_FC) ≥ 1’ as the threshold for DEG selection. For pathway enrichment analysis, DEGs were mapped to the terms in the KEGG database by using the KEGG Orthology Based Annotation System (KOBAS) [40]. Significantly enriched pathways with respect to DEGs were identified with the criterion of a corrected *P*-value ≤0.05.

### Quantitative RT-PCR analysis

For quantitative RT-PCR of mRNAs, 2 μg DNase I-treated total RNA was used to synthesize cDNA by M-MLV (Promega). qRT-PCR was performed using KAPA™ SYBR® FAST qPCR kits (Kapa Biosystems, Woburn, MA) on a StepOne Plus Real Time PCR System (Applied Biosystems, Foster City, USA) according to the manufacturer’s instruction. Products were verified by melting curve analysis. Quantification was achieved by normalizing the number of target transcripts copies to the reference *GhACTIN* gene using the comparative ΔΔCt method [41]. All reactions were performed with three biological replicates. Primers used in all quantitative RT-PCR experiments are listed in Additional file 2: Table S1.

### ABA, proline and malondialdehyde (MDA) content quantification

Stem segment which is 7–12 cm below the floral head was used for detection of content of ABA, proline and MDA. The ABA content determination was performed on an HPLCMS/MS (LCMS-8040 system, Shimadzu) according to a method described previously [42]. The proline level was measured using the acid ninhydrin method [43, 44]. The measurement of MDA was conducted as described by Heath and Packer (1968) and Deng et al. (2011) [44, 45].

## Results

### Transcriptome sequencing and de novo assembly

Three cDNA libraries were constructed using equal amounts of RNA extracted from stems of *G. hybrida* at stem bending stage 0, 2, and 4, respectively. To characterize the *G. hybrida* transcriptome, the cDNA library was subjected to PE read sequencing using the Illumina HiSeq2000 platform. After filtering out primer and adapter sequences and the low-quality reads, we obtained a total of 304,982,214 clean reads, approximately 36.5 Gb of data bulk, with an average GC content of 45.0% (Table 1). These high-quality reads were then assembled to generate 96,492 unigenes, which accounted for 63,846,992 nucleotides. The length of unigenes was varied from 201 to 15,726 bp, with an average of 662 bp. The N50 was 1,066 bp. The sequence length distribution of unigenes is presented in Additional file 1: Figure S2. Among these unigenes, 43,261 (44.8%) were longer than 400 bp, 16,807 (17.4%) were longer than 1000 bp, and 9,135 (9.5%) were longer than 1600 bp.

**Table 1:** Sequence statistics of the *G. hybrida* transcriptome

Total number of reads	304,982,214
Number of reads at Stage 0	90,783,368
Number of reads at Stage 2	106,570,262
Number of reads at Stage 4	107,628,584
Total number of unigenes	96,492
Total nucleotides of unigenes (bp)	63,846,992
Mean length of unigenes (bp)	662
Largest unigenes (bp)	15,726
Smallest unigenes (bp)	201
N50	1,066

### Functional annotation of unigenes

**Fig. 1:**
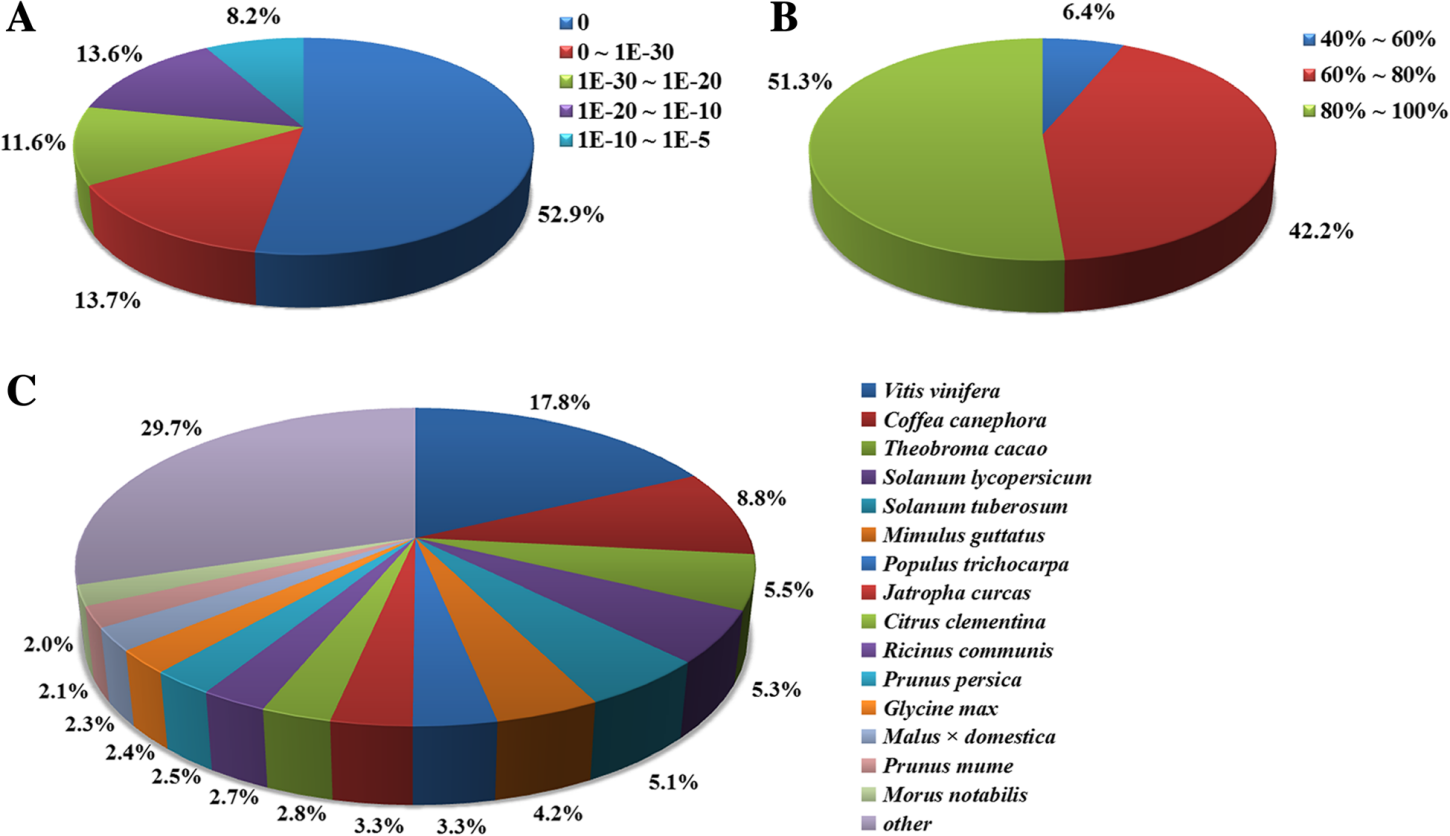
Characteristics of homology search of unigene sequences aligned by BlastX to the NR database. **a** E-value distribution of BlastX hits for each unique sequence with an E-value threshold of 1.0E−5. **b** Similarity distribution of top BlastX hits for each unigene. **c** Species distribution of the top BlastX hits for each unigene sequence with a cut-off E-value of 1.0E−5

The assembled unigene sequences were annotated through homologous search against the public databases using the BlastX with a cut-off E-value of 10^− 5^. In total, 34,166 unigenes (35.4% of the total 96,492 unigenes) had homologs in at least one of the public databases that we searched, including NR, Swissprot protein database, String protein database, Pfam protein families database, KEGG database, GO database, the COGs database and Eukaryotic Orthologous Groups (KOGs) database (Table 2). Among them, 31,805 (33.0%) and 20,706 (21.5%) unigenes had significant hits in the NR and Swissprot non-redundant protein database, respectively. Unigenes annotated by the NR database were analyzed (Fig. 1). The E-value distribution results showed that 33.4% of the homologs ranged between 1.0E^− 5^ to 1.0E^− 30^, while a majority of the sequences (66.6%) had the E-value less than 1.0E^− 30^, indicating strong homology (Fig. 1a). In terms of similarity distribution, 51.3% of the matches ranged from 80 to 100% similarity as reported in the BlastX results, while 42.2% of the matches were of similarity ranging from 60 to 80%, and only 6.4% had less than 60% similarity with the corresponding gene sequence (Fig. 1b). The species that exhibited the best BlastX matches was *Vitis vinifera* (17.8%). The second was *Coffea canephora*, which showed 8.8% homology with *G. hybrida* (Fig. 1c).

**Table 2:** List of *G. hybrida* transcriptome annotations

Public database	No. of unigene hit	Percentage (%)
NR	31,805	33.0
Swissprot	20,706	21.5
String	14,243	14.8
Pfam	18,202	18.9
KEGG	10,911	11.3
COG	10,085	10.5
KOG	10,313	10.7
GO	18,253	18.9

*NR* Non-redundant protein sequence database

*SwissProt* Swiss-Prot protein sequence database

*String* String protein sequence database

*Pfam* Pfam protein families database

*KEGG* Kyoto Encyclopedia of Genes and Genomes

*COG* Cluster of Orthologous Groups of proteins

*KOG* Eukaryotic Orthologous Groups

*GO* Gene Onotology database

**Fig. 2:**
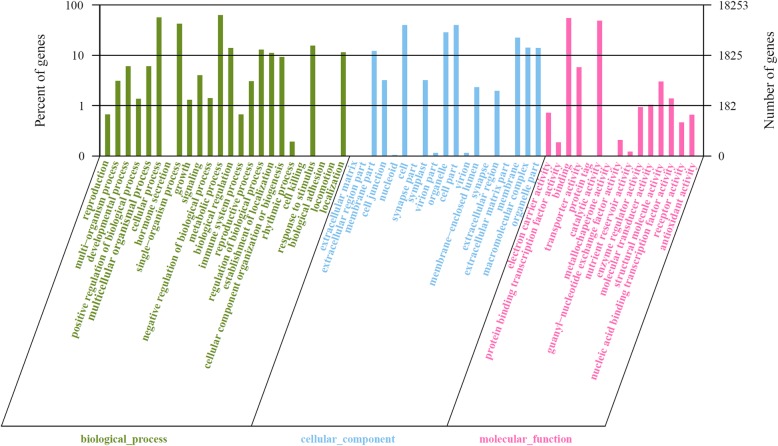
GO assignments for the *G. hybrida* transcriptome. Results are summarized under three main GO categories: biological process, cellular component and molecular function. The left y-axis indicates the percentage of a specific category of genes in each main category, while the right y-axis indicates the number of genes in the same category

GO terms was used to classify functions of the annotated genes. Using the Blast2GO program, 18,253 unigenes were categorized into three main GO trees, including biological process, cellular component and molecular function, and some of them belonged to one or more of the three categories (Fig. 2). Among the annotated unigenes, 15,099 (82.7%) were assigned in the molecular function category, 14,158 (77.6%) in the biological process category, and 9,117 (49.9%) in the cellular component category, whereas 6,489 (35.6%) in all three categories. The three main categories were further classified into 58 functional groups. In the molecular function category ‘binding’ (10,041) is the most dominant cluster. Other two highly abundant groups are ‘catalytic activity’ (8,890), and ‘transporter activity’ (1,052). In the cellular component category, the GO terms ‘cell’ (7,281), ‘cell part’ (7,281), ‘organelle part’ (5,216), and ‘membrane’ (4,069) predominated. In the biological process category ‘metabolic process’ (11,498) represented the most abundant group, which was consistent with the fact that our transcriptome data were derived from gerbera plants during the post-harvest senescence process. The other prominent GO terms in this category included ‘cellular process’ (10,342), ‘single-organism process’ (7,683), and an interesting group ‘response to stimulus’ (2,840).

**Fig. 3:**
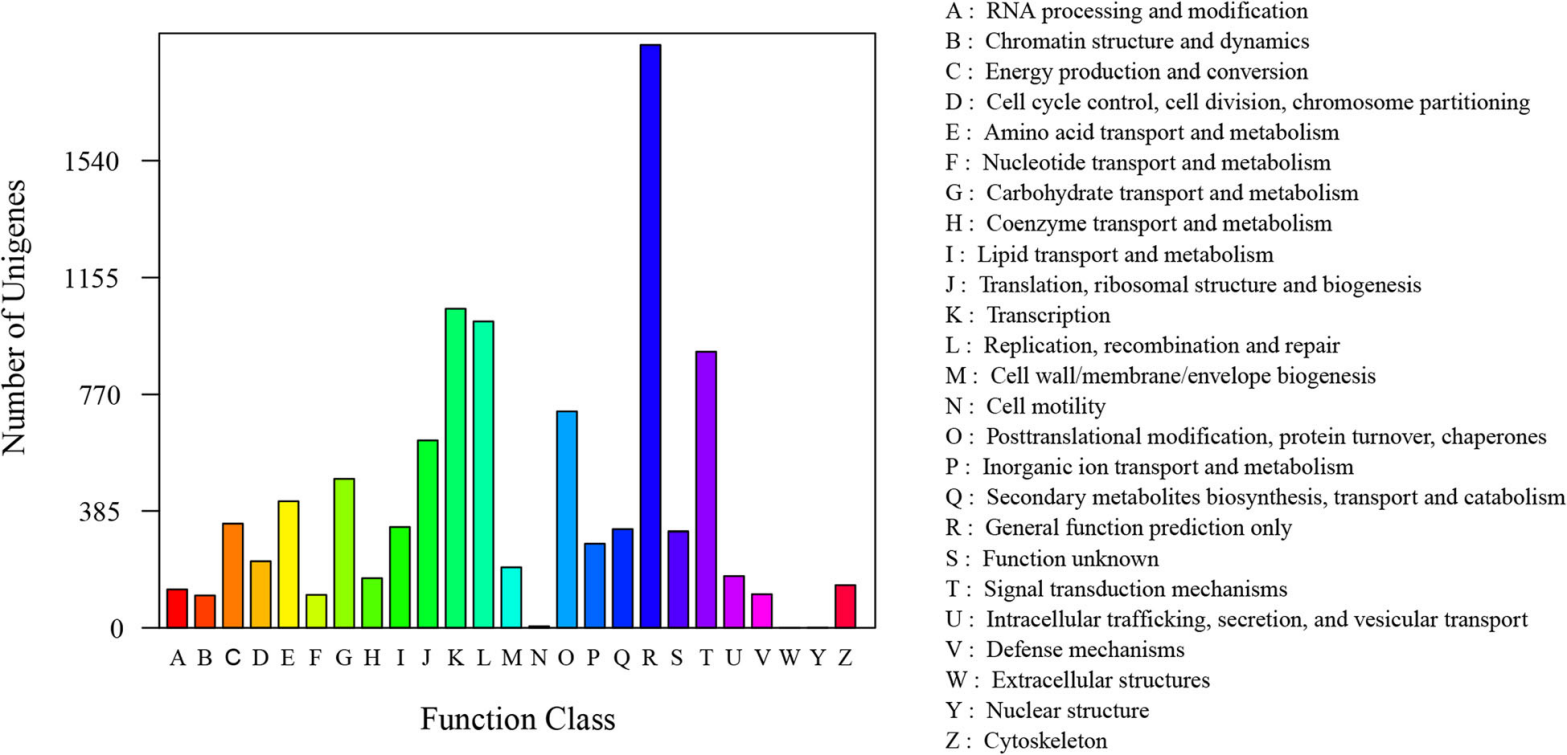
Distribution of genes in the transcriptome with COG functional classification

We also performed phylogenetic classification using COG database. Based on sequence homology, 10,085 unigenes were matched and grouped into 25 functional classes (Fig. 3). The top two clusters with the highest percentage were ‘General function prediction only’ (1,921) and ‘Transcription’ (1,052), which represented 19.0 and 10.4%, respectively. The next three were ‘replication, recombination and repair’ (1,010; 10.0%), ‘signal transduction mechanisms’ (910; 9.0%), and ‘posttranslational modification, protein turnover, chaperones’ (714; 7.1%). Moreover, 318 (3.2%) unigenes were assigned to the ‘function unknown’ category. In addition, few unigenes were grouped in the ‘extracellular structures’ (0; 0.0%) and ‘nuclear structure’ (1; 0.0%) categories.

We mapped the annotated sequences onto the KEGG database. Totally 10,911 unigenes were functionally assigned to 164 KEGG pathways (Additional file 2: Table S2). Among them, the ‘metabolic pathways’ was the most dominant group (2,458; 22.5%), followed by ‘Biosynthesis of secondary metabolites’ (1,192; 10.9%) and ‘Ribosome’ (466; 4.3%).

### Identification of DEGs

**Fig. 4:**
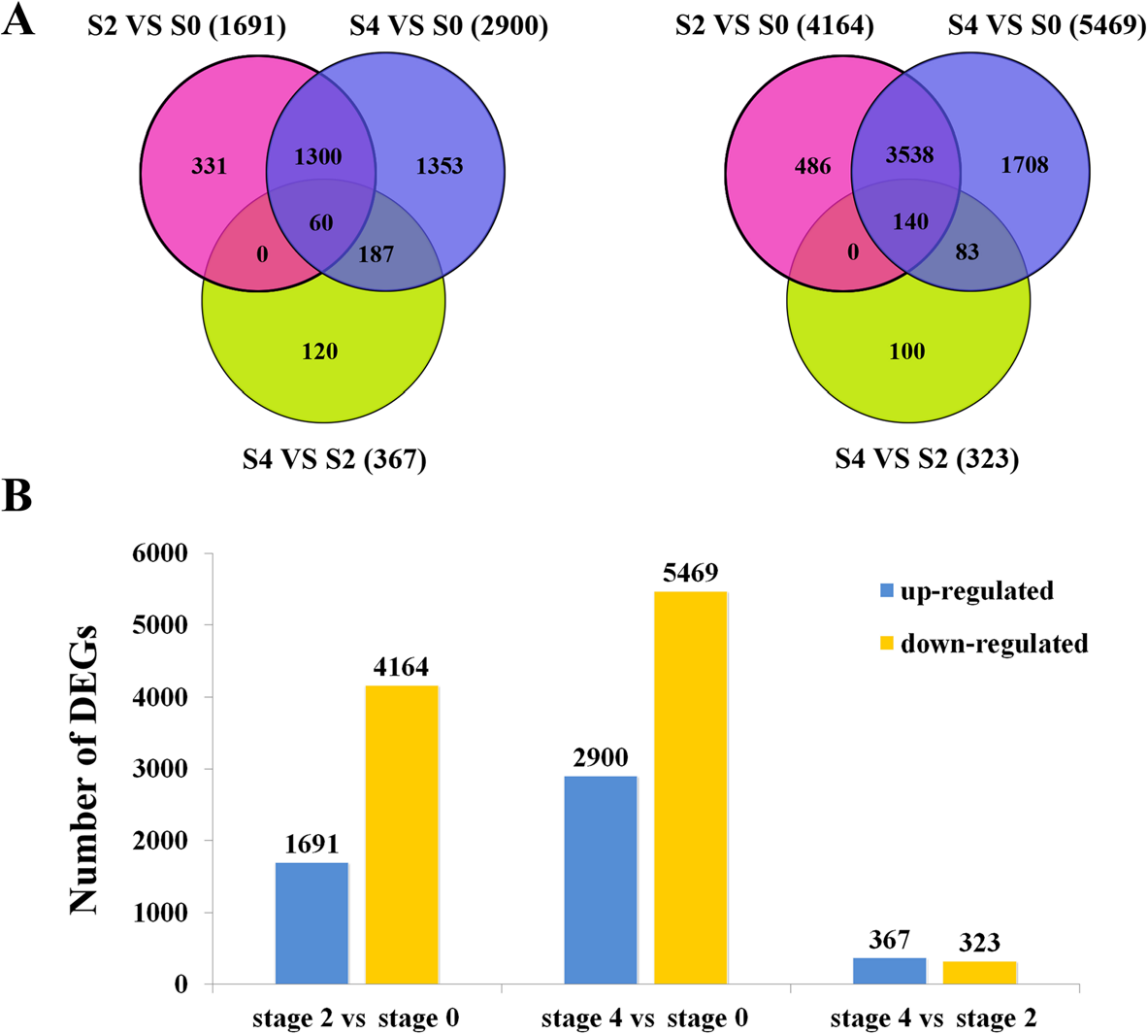
DEGs for *G. hybrida* during stem bending process. **a** Venn maps of up-regulated (left) and down-regulated (right) genes. S, stage. **b** The number of up-regulated and down-regulated genes compared between different bending stages. FDR ≤ 0.05 and |log_2_FC| ≥ 1were used as the threshold to judge the significance of gene expression difference

To gain a global view of transcript expression for *G. hybrida* during stem bending process, we analyzed the genome-wide expression by sequencing. Illumina reads from each library were mapped onto the assembled transcriptome database. The expression of each gene was calculated based on the numbers of reads mapping onto the transcripts. The correlation coefficients of the six samples were also analyzed and listed in Additional file 3. A total of 9,406 significantly changed unigenes (FDR ≤ 0.05 and |log_2_FC| ≥ 1), including 3,351 up-regulated genes and 6,055 down-regulated genes, were detected between three different libraries. As shown in Fig. 4, comparison of gene expression between stage 2 and stage 0 (stage 2 vs stage 0) showed that 5,855 genes were significantly differently expressed, with 1,691 up-regulated and 4,164 down-regulated and log_2_FC value ranging from − 11.7 to 10.8 (Additional file 4). For stage 4 vs stage 0, there were 8,369 DEGs with 2,900 up-regulated and 5,469 down-regulated and log_2_FC value ranging from − 12.1 to 10.9. For stage 4 vs stage 2, 690 genes were significantly differently expressed, with 367 up-regulated and 323 down-regulated and log_2_FC value changing from − 11.1 to 8.9. In the two comparison groups of stage 2 vs 0 and stage 4 vs 0, 1,360 up-regulated and 3,678 down-regulated DEGs in common were identified; while in all the three comparison groups, only 60 up-regulated and 140 down-regulated DEGs in common were found (Fig. 4a).

**Fig. 5:**
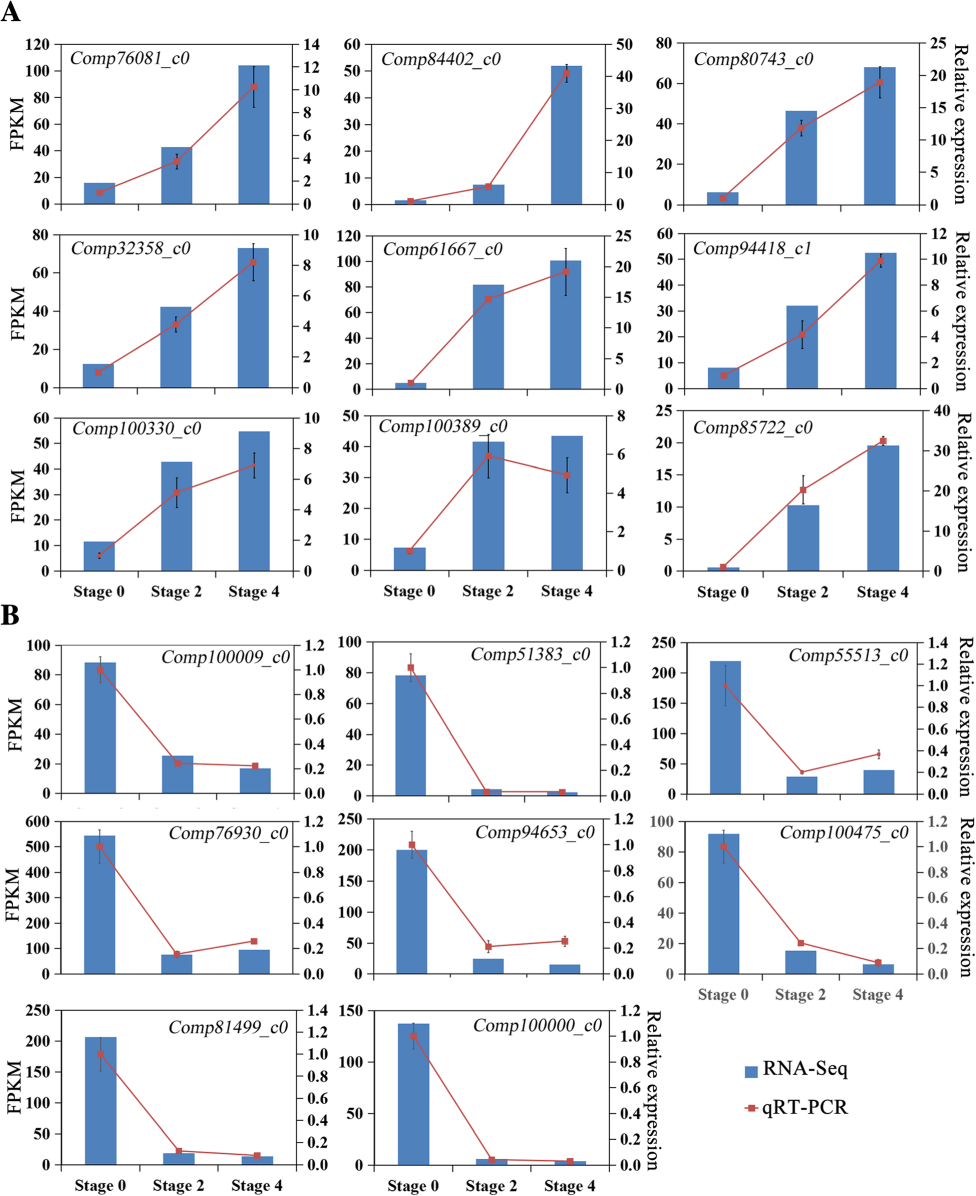
Verification of RNA-seq results by qRT-PCR. Seventeen DEGs with significantly altered expression pattern during stem bending, including 9 up-regulated (**a**) and 8 down-regulated (**b**), were randomly selected and expression level of DEGs between RNA-seq and qRT-PCR were compared. The Y-axises show corresponding expression data of RNA-seq (blue histogram, left) and the relative gene expression levels analyzed by qPCR (red lines, right). The X-axis represents the bending stage. Primers for qRT-PCR are listed in Additional file 2: Table S1

To further validate the RNA-seq data, we performed qRT-PCR on seventeen randomly selected DEGs, including nine up-regulated and eight down-regulated during stem bending process in the DEG dataset (Fig. 5). The results showed that expression patterns of the DEGs were quite similar between RNA-seq and qRT-PCR analyses, though the FC values were varied to some extent. This result indicated the high consistency between the two analysis techniques, supporting the reliability of the RNA-Seq analysis.

### Pathways and biological process enrichment analysis of DEGs

To explore the biological functions of the DEGs, we mapped the DEGs to terms in KEGG database by KOBAS, with a view to identify significantly enriched metabolic pathways or signal transduction pathways in DEGs when compared with the whole genome background. Results showed that 23, 32 and 13 pathways were significantly enriched (corrected *P*-value ≤0.05), respectively, in the comparisons of stem bending stage 2 vs stage 0, stage 4 vs stage 0, and stage 4 vs stage 2. The top 10 pathways in each group were indicated in Table 3. Notably, specific enrichment was observed in ‘phenylpropanoid biosynthesis’, ‘phenylalanine metabolism’, ‘starch and sucrose metabolism’, ‘plant hormone signal transduction’, and ‘amino sugar and nucleotide sugar metabolism’ in both comparisons of stage 2 vs stage 0 and stage 4 vs stage 0. In the stage 4 vs stage 2 group, the ‘biosynthesis of unsaturated fatty acids’, ‘phenylpropanoid biosynthesis’, ‘phenylalanine metabolism’, ‘monoterpenoid biosynthesis’, and ‘nitrogen metabolism’ were significantly identified as the top five enriched pathways. In particular, four pathways, including ‘phenylpropanoid biosynthesis’, ‘phenylalanine metabolism’, ‘starch and sucrose metabolism’, and ‘plant hormone signal transduction’, were commonly enriched in all the three comparison groups.

**Table 3:** Significantly enriched pathways involving DEGs during stem bending

	Pathway	Pathway ID	Number of all genes with pathway annotation (10,911)	Number of DEGs with pathway annotation (4,653)	Corrected *P*-Value
stage 2 vs stage 0	Phenylpropanoid biosynthesis	ko00940	146 (1.3%)	70 (1.5%)	0.00E+ 00
	Phenylalanine metabolism	ko00360	109 (1.0%)	47 (1.0%)	5.81E-10
	Starch and sucrose metabolism	ko00500	238 (2.2%)	76 (1.6%)	9.83E-09
	Plant hormone signal transduction	ko04075	316 (2.9%)	93 (2.0%)	9.83E-09
	Amino sugar and nucleotide sugar metabolism	ko00520	157 (1.4%)	53 (1.1%)	4.97E-07
	Stilbenoid, diarylheptanoid and gingerol biosynthesis	ko00945	24 (0.2%)	15 (0.3%)	8.22E-06
	Flavonoid biosynthesis	ko00941	34 (0.3%)	18 (0.4%)	1.46E-05
	Pentose and glucuronate interconversions	ko00040	99 (0.9%)	34 (0.7%)	7.52E-05
	Cyanoamino acid metabolism	ko00460	54 (0.5%)	22 (0.5%)	1.68E-04
	Gap junction	ko04540	52 (0.5%)	21 (0.5%)	2.74E-04
stage 4 vs stage 0	Plant hormone signal transduction	ko04075	316 (2.9%)	120 (2.6%)	0.00E+ 00
	Phenylpropanoid biosynthesis	ko00940	146 (1.3%)	78 (1.7%)	0.00E+ 00
	Phenylalanine metabolism	ko00360	109 (1.0%)	56 (1.2%)	4.68E-13
	Starch and sucrose metabolism	ko00500	238 (2.2%)	87 (1.9%)	1.03E-09
	Amino sugar and nucleotide sugar metabolism	ko00520	157 (1.4%)	56 (1.2%)	8.01E-06
	Stilbenoid, diarylheptanoid and gingerol biosynthesis	ko00945	24 (0.2%)	16 (0.3%)	1.01E-05
	Cysteine and methionine metabolism	ko00270	125 (1.1%)	46 (1.0%)	2.46E-05
	Flavonoid biosynthesis	ko00941	34 (0.3%)	19 (0.4%)	3.22E-05
	Gap junction	ko04540	52 (0.5%)	24 (0.5%)	1.08E-04
	Cyanoamino acid metabolism	ko00460	54 (0.5%)	24 (0.5%)	2.24E-04
stage 4 vs stage 2	Biosynthesis of unsaturated fatty acids	ko01040	65 (0.6%)	14 (0.3%)	1.23E-11
	Phenylpropanoid biosynthesis	ko00940	146 (1.3%)	13 (0.3%)	3.90E-06
	Phenylalanine metabolism	ko00360	109 (1.0%)	10 (0.2%)	6.47E-05
	Monoterpenoid biosynthesis	ko00902	10 (0.1%)	3 (0.1%)	4.78E-03
	Nitrogen metabolism	ko00910	68 (0.6%)	6 (0.1%)	4.78E-03
	Metabolism of xenobiotics by cytochrome P450	ko00980	53 (0.5%)	5 (0.1%)	9.74E-03
	Plant hormone signal transduction	ko04075	316 (2.9%)	12 (0.3%)	1.40E-02
	Starch and sucrose metabolism	ko00500	238 (2.2%)	10 (0.2%)	1.52E-02
	Drug metabolism - cytochrome P450	ko00982	46 (0.4%)	4 (0.1%)	2.92E-02
	Ubiquinone and other terpenoid-quinone biosynthesis	ko00130	48 (0.4%)	4 (0.1%)	3.13E-02

Then we identified GO terms that were significantly enriched during stem bending and focused on the ones that belong to the biological process category (Additional file 5). As expected, GO terms such as ‘phenylpropanoid biosynthetic and catabolic process’, ‘L-phenylalanine metabolic process’, ‘starch metabolic process’ and ‘sucrose metabolic process’ were highly enriched in both stage 2 vs stage 0 and stage 4 vs stage 0 groups, which coincided with the KEGG result. ‘Lignin catabolic process’, ‘cell wall modification’, ‘pectin catabolic process’, and ‘cellulose metabolic process’, were also enriched in this process, showing that cell wall firmness of the stem may decline during the stem bending. Furthermore, GO terms related to responses to various types of abiotic stresses including response to osmotic stress, oxidative stress, salt stress, light stimulus and temperature stimulus were enriched in this process, suggesting that some of these stresses may occur in stem during the vase life of gerbera and induce stem bending. And the crosstalk of different stress responses in gerbera may also exist as those reported in other species [46]. In addition, it was noticed that two GO terms, ‘response to hormone stimulus’ and ‘response to auxin stimulus’, were also enriched in the stem bending process. The KEGG and GO enrichment analysis suggested that regulation of hormone signaling, maintenance of energy and carbon supply, as well as stabilization of cell protein and structures may be related to stem bending of gerbera plants.

### Expression of genes involved in hormone signaling pathways

An objective of our work was to study the expression of DEGs involved in hormone signaling pathway. In total, 93, 120 and 12 DEGs identified in comparisons of stage 2 vs stage 0, stage 4 vs stage 0, and stage 4 vs stage 2, respectively, were found to be associated with plant hormone signal transduction pathways (Table 3), suggesting that the expression of many DEGs involved in the hormone signaling pathways were changed in the early stage of stem bending, while varied little between bending stage 2 and stage 4.

Expression of many unigenes involving in signaling of phytohormones, such as auxin, cytokinins (CTK), gibberellin (GA), abscisic acid (ABA), ethylene (ETH), brassinosteroids (BR), and salicylic acid (SA) showed significantly changes during the stem bending process. The DEGs with substantial changes (|log_2_FC| ≥ 2 at least at one stage and FDR ≤ 0.05) were listed in Table 4. We found that only a small amount of DEGs were identified in signal transduction of GA, BR and SA, indicating these hormones may be not involved in this process. Conversely, auxin responsive genes accounted for a large proportion of the total DEGs. However, our data showed that expression of most genes of *auxin*/*indole-3-acetic acid* (*Aux*/*IAA*), the *GH3*, and the *small auxin-up RNA* (*SAUR*), the three major classes of early or primary auxin response genes, was down-regulated in different degree during stem bending. Thus, more evidences are needed to investigate whether auxin is necessary for stem bending. Then we focused on the ETH and ABA signaling pathways because of their involvement in the regulation of senescence process of many plants. In ETH signal transduction pathway, genes of two components, including ethylene receptor (ETR) and ethylene-responsive transcription factor (ERF) [47], differently expressed during stem bending. Gene encoding ETR was induced greatly at stage 2 and stage 4, while genes related to *ERF*, which is an early ethylene responsive gene, showed different expression pattern at stage 2 and 4, including three up-regulated and six down-regulated, indicating that the role of ethylene in the stem bending may also be not essential. Remarkably, all DEGs involved in ABA signaling pathway, except comp76549_c0, showed increased expression during the stem bending. For example, three genes encoding *protein phosphatase 2C* (*PP2C*) were induced at stage 2 and the expression level kept high at stage 4, especially comp112827_c0, whose expression was strongly increased at both stem bending stages. Two genes of *serine*/*threonine-protein kinase SAPK3-like* showed the similar up-regulated expression pattern. In addition, the gene comp76549_c0 with decreased transcript abundance encodes protein of ABA receptor PYR/PYLs, which function in the control of ABA signaling by inhibiting PP2Cs [48]. Such data suggested that ABA may play an important role in the stem bending of gerbera.

**Table 4:** DEGs related to plant hormone signal transduction pathway during stem bending

Gene ID	Putative function	Stage 2 vs Stage 0		Stage 4 vs Stage 0	
		FDR	log_2_FC	FDR	log_2_FC
Auxin signal transduction					
comp108122_c0	auxin influx carrier protein (AUX1, LAX)	8.61E-23	−4.08	4.62E-57	−6.79
comp107506_c0	auxin influx carrier protein (AUX1, LAX)	1.02E-39	−4.81	6.38E-106	−5.10
comp94670_c0	auxin influx carrier protein (AUX1, LAX)	7.49E-71	−2.89	0.00E+ 00	− 3.68
comp99743_c0	auxin-responsive protein AUX/IAA	2.27E-43	2.36	3.60E-186	2.37
comp99899_c0	auxin-responsive protein AUX/IAA	9.62E-114	4.00	2.37E-217	3.78
comp87612_c0	auxin-responsive protein AUX/IAA	4.27E-06	−2.87	8.83E-23	−3.73
comp74586_c0	auxin-responsive protein AUX/IAA	2.78E-05	−2.37	8.94E-20	−3.34
comp87358_c0	auxin-responsive protein AUX/IAA	4.56E-26	−2.73	5.16E-136	−3.47
comp89152_c0	auxin-responsive protein AUX/IAA	1.37E-44	−2.50	6.35E-235	−3.35
comp65420_c0	auxin-responsive protein AUX/IAA	6.08E-32	−2.71	1.90E-130	−3.14
comp99733_c0	auxin-responsive protein AUX/IAA	9.37E-40	−2.10	7.74E-213	−2.62
comp66980_c0	auxin-responsive protein AUX/IAA	1.74E-26	−3.15	3.50E-107	−3.40
comp83901_c0	auxin-responsive protein AUX/IAA	7.28E-08	−3.20	6.12E-14	−3.59
comp32212_c0	auxin-responsive protein AUX/IAA	2.83E-23	−3.33	8.97E-80	−3.48
comp85722_c0	auxin response factor (ARF)	2.89E-33	4.00	6.69E-114	4.95
comp71781_c0	auxin response factor (ARF)	8.92E-10	−2.21	3.63E-16	−2.42
comp89590_c0	Auxin-responsive GH3 family protein (GH3)	2.28E-22	−2.32	1.83E-99	−2.77
comp77123_c0	SAUR family protein	1.88E-40	4.97	1.75E-75	4.91
comp87079_c0	SAUR family protein	1.75E-32	2.05	2.33E-79	2.12
comp97116_c0	SAUR family protein	1.32E-35	−3.10	1.83E-179	−3.39
comp80117_c0	SAUR family protein	1.46E-25	−5.52	4.12E-59	−5.67
comp84807_c0	SAUR family protein	5.24E-18	−3.91	1.31E-42	−5.54
comp85286_c0	SAUR family protein	8.63E-17	−3.06	2.49E-40	−4.54
comp47517_c0	SAUR family protein	1.31E-18	−5.01	1.31E-38	−4.70
CTK signal transduction					
comp321451_c0	cytokinin receptor (CRE1)	4.14E-03	−6.26	2.32E-06	−6.69
comp79050_c0	type A response regulator (ARR-A)	3.49E-12	−3.32	1.89E-14	−2.44
comp36292_c0	type A response regulator (ARR-A)	2.68E-59	−4.07	3.28E-169	−4.00
comp86047_c0	type A response regulator (ARR-A)	3.50E-05	−2.48	1.48E-18	−3.23
comp107220_c0	type A response regulator (ARR-A)	1.48E-10	−2.11	8.65E-45	−2.47
Gibberellin signal transduction					
comp94395_c0	DELLA protein	2.51E-07	−2.32	1.61E-06	−1.58
comp34284_c0	F-box protein GID2	3.10E-04	2.06	8.81E-13	2.57
ABA signal transduction					
comp76549_c0	abscisic acid receptor PYR/PYL family	7.87E-12	−1.06	6.93E-271	−4.24
comp112827_c0	protein phosphatase 2C (PP2C)	1.08E-05	7.17	4.00E-25	8.69
comp69932_c0	protein phosphatase 2C (PP2C)	2.23E-06	5.36	3.53E-49	7.18
comp82660_c0	protein phosphatase 2C (PP2C)	2.92E-07	0.89	1.97E-133	2.22
comp126799_c0	serine/threonine-protein kinase SAPK	2.46E-02	5.98	5.70E-06	6.47
comp117023_c0	serine/threonine-protein kinase SAPK	7.22E-02	2.77	2.79E-07	3.23
Eth signal transduction					
comp94418_c0	ethylene receptor (ETR)	1.80E-27	1.83	8.21E-226	2.29
comp61667_c0	ethylene-responsive transcription factor (ERF)	1.23E-87	4.02	0.00E+ 00	4.18
comp32358_c0	ethylene-responsive transcription factor (ERF)	7.67E-18	1.82	2.16E-111	2.38
comp85680_c0	ethylene-responsive transcription factor (ERF)	1.35E-30	2.23	2.85E-147	3.05
comp74437_c0	ethylene-responsive transcription factor (ERF)	2.23E-02	−1.41	5.41E-18	−3.97
comp80312_c0	ethylene-responsive transcription factor (ERF)	2.86E-24	−2.68	2.30E-24	−1.66
comp55944_c0	ethylene-responsive transcription factor (ERF)	3.54E-17	−6.27	4.06E-37	−9.23
comp191038_c0	ethylene-responsive transcription factor (ERF)	2.04E-07	−2.17	1.32E-16	−3.27
comp69820_c0	ethylene-responsive transcription factor (ERF)	2.02E-09	−5.35	2.84E-20	− 5.31
comp33734_c0	ethylene-responsive transcription factor (ERF)	3.87E-14	−2.36	5.33E-95	−5.77
Brassinosteroid signal transduction					
comp119011_c0	brassinosteroid resistant 1/2	2.03E-03	−2.29	1.25E-02	−1.35
SA signal transduction					
comp80158_c0	BZIP transcription factor family protein (TGA)	7.98E-09	−3.61	3.49E-15	−3.34

DEGs with |log_2_FC| ≥ 2 at least at one stage and FDR ≤ 0.05 were included

**Fig. 6:**
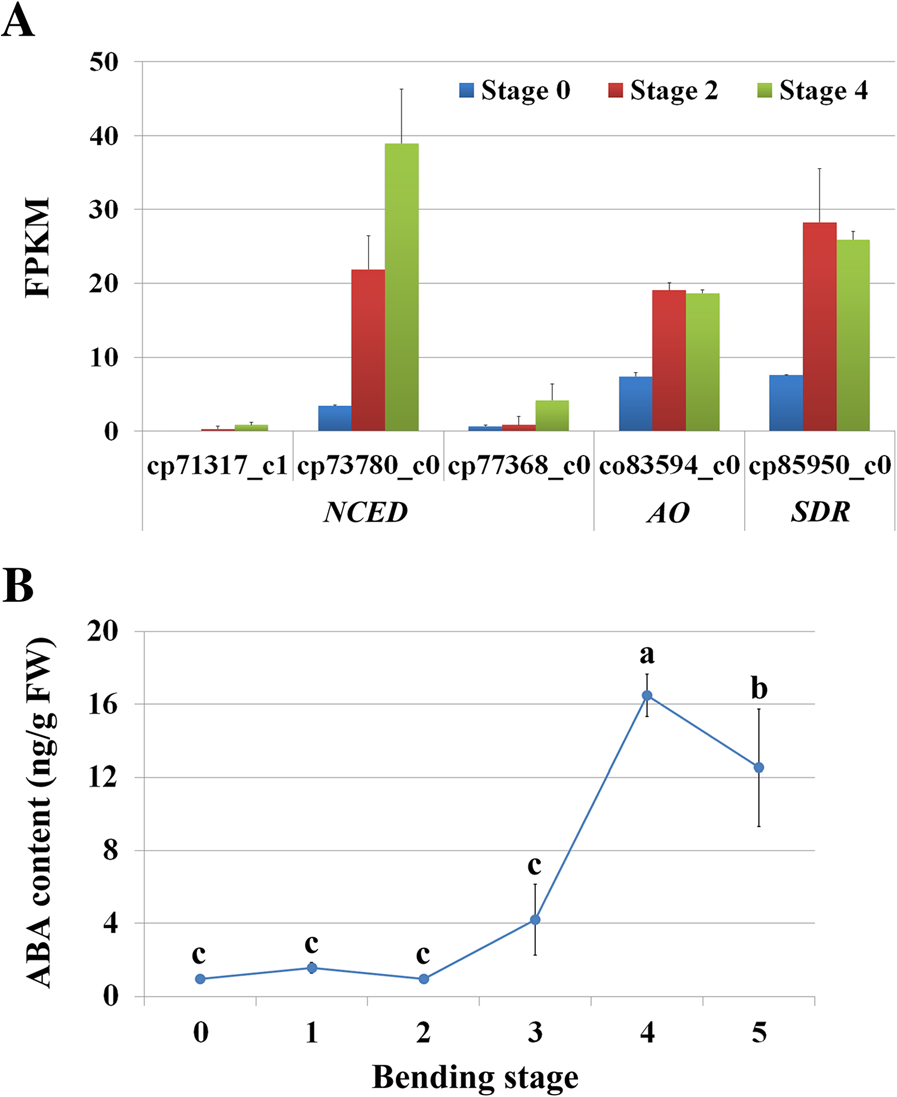
Expression of ABA biosynthesis-related genes and changes of endogenous ABA level of bent stem segment of gerbera during stem bending. **a** Expression of ABA biosynthesis-related genes detected in the transcriptome data. FPKM: fragments per kilobase of exon model per million mapped reads; *NCED*, 9-cis-epoxycarotenoid dioxygenase; *AO*, aldehyde oxidase; *SDR*, short-chain dehydrogenase/reductase. **b** Mean levels of ABA content of bent stem segments at different bending stages. Values are means ± SD (*n* = 3). Lowercase letters indicate significant differences of ABA levels among different bending stages (one-way ANOVA, Duncan’s multiple range test)

To further investigate the involvement of ABA in this process, we analyzed the expression of key genes of ABA biosynthesis. In total, we found that five DEGs, including three genes encoding 9-*cis*-epoxycarotenoid dioxygenase (NCED), one encoding aldehyde oxidase (AO), and one encoding short-chain dehydrogenase/reductase (SDR)*,* were up-regulated during stem bending (Fig. 6a). Then we also tested the content of endogenous ABA in the stem segment (7–12 cm below the floral head) during stem bending. Our result showed that ABA level was low at bending stage 0 to 2, and slightly increased at stage 3. It should be noticed that the content of ABA rose dramatically and reached to the highest value at stage 4. Its content in stage 4 sample was nearly 4 times higher than in stage 3 sample. Then the level went down from stage 4 to stage 5, when the stem is nearly broken (Fig. 6b). The increase of endogenous ABA content in bent stem segment along with the development of stem-bending suggested that ABA may be one of the major regulators during stem bending of gerbera.

### Analysis of transcription factors (TFs) during the stem bending process

**Fig. 7:**
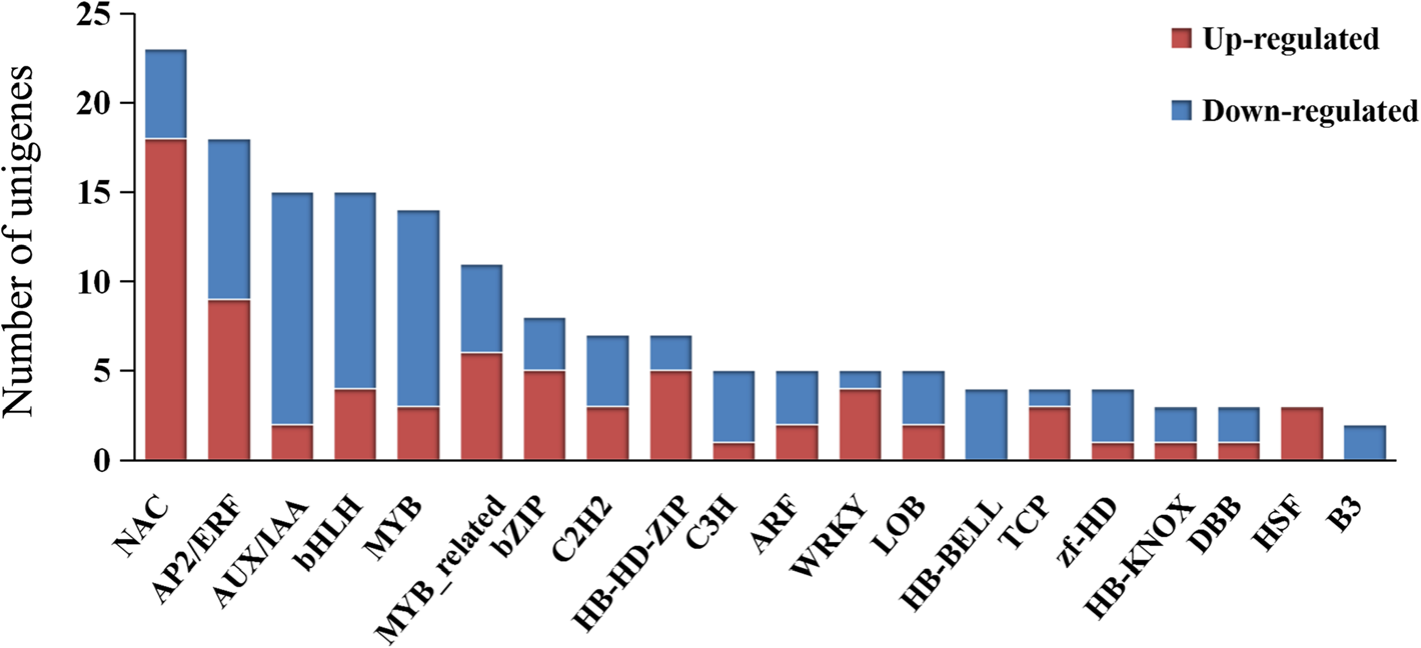
Number of unigenes annotated as TF. The top 20 families of TF are listed. Red and blue columns indicate unigenes up-regulated and down-regulated during the stem bending process, respectively

TFs are important upstream regulators of plant development and senescence. In the present study, we identified a total of 211 DEGs which are annotated as TFs through homologous search of DEGs against the Plant Transcription Factor Database and iTAK database using the BlastX (E-value <1e^− 5^). These TFs were classified into 50 families and the top 20 TF families were indicated in Fig. 7. Among them the NAC family constituted the largest group, containing 23 unique transcripts (18 up-regulated and 5 down-regulated), followed by the AP2/ERF (9 and 9), AUX/IAA (2 and 13), bHLH (4 and 11), MYB (3 and 11), MYB-related (6 and 5), bZIP (5 and 3), and C2H2 (3 and 4) families. These eight families accounted for about half of the listed TFs.

We focused on the TFs with increased expression during stem bending. As a result, a total of 98 up-regulated putative TFs were identified (Additional file 6). It can be observed that the expression pattern varied among the TFs. Expression of about 84.7% of the induced TFs increased shortly after bending occurs, i.e. stage 2. The majority of TFs at stage 4 kept similar expression level to those at stage 2, only 20.4% TFs expressed on a significantly higher level at stage 4 than stage 2. Interestingly, we found that 5 unigenes of *DREB*/*CBF* genes, which belong to the AP2 family and function in regulating ABA-independent gene expression in response to drought [49], were up-regulated during the stem bending process in gerbera. The biggest increase of expression was found in comp45797_c0, which was annotated as a *DREB1A* gene and had a log_2_FC of 3.77 and 4.47 in the comparisons of bending stage 2 vs stage 0 and stage 4 vs stage 0, respectively. We also found two transcripts encoding NF-YA subunits, which are members of plant NF-Y (Nuclear Factor Y), an important regulator that can coordinate plant responses to drought stress, showed increased expression during the stem bending. These results collectively suggested that the stem may suffer from a medium water stress during the stem bending process in gerbera.

### Expression of genes involved in responses to osmotic and oxidative stresses

To further investigate if stress response happens in the stem during the stem bending process, we examined the expression of genes involved in responses to stresses. Among the stress responses category (GO:0006950), it was noticed that osmotic stress (GO:0006970) and oxidative stress (GO:0006979) were significantly enriched at bending stage 2 and 4 when compared to stage 0. Both up- and down-regulation of gene expression was observed, and the expression of genes changed over time of stem bending. We focused on the up-regulated genes during the stem bending process and listed them in Table 5. Among all DEGs enriched in osmotic stress category, 9 genes were quickly induced at stage 2, and 14 showed significantly differential expression at stage 4 when compared to stage 0. The top three genes significantly induced during the stem bending process were homeobox-leucine zipper protein ATHB 12-like protein (comp170518_c0), mitochondrial phosphate transporter 3 (comp88289_c0), and serine/threonine-protein kinase SAPK1 (comp116208_c0). For genes enriched in the oxidative stress gene category, 24 DEGs, which were differently expressed in comparison of stage 2 vs stage 0 and/or stage 4 vs stage 0, were identified. The top three genes significantly up-regulated were vacuolar iron transporter homolog (comp74749_c0), peroxidase (comp54647_c0), and cationic peroxidase (comp88411_c0).

**Table 5:** DEGs related to osmotic and oxidative stresses and proline biosynthetic pathway during stem bending

Gene ID	Putative function	Stage 2 vs Stage 0		Stage 4 vs Stage 0	
		FDR	log_2_FC	FDR	log_2_FC
Osmotic stress related genes					
comp170518_c0	Homeobox-leucine zipper protein ATHB 12-like			1.63E-09	**7.49**
comp88289_c0	Mitochondrial phosphate transporter 3;1	5.60E-03	**2.73**	6.78E-06	**2.61**
comp116208_c0	Serine/threonine-protein kinase SAPK 3			8.60E-05	**3.01**
comp46146_c0	DEAD-box ATP-dependent RNA helicase 53			4.94E-02	**1.34**
comp73954_c0	SGF29 tudor-like domain	4.51E-03	**1.28**	3.42E-02	0.87
comp56410_c0	Mitochondrial HSO70 isoform	3.56E-15	**1.23**	1.10E-38	**1.16**
comp87549_c0	Lipocalin / cytosolic fatty-acid binding protein	3.74E-16	**1.23**	1.38E-86	**1.23**
comp84431_c0	Mitochondrial HSO70 isoform	8.44E-07	**1.12**	5.20E-24	**1.22**
comp97921_c0	3-ketoacyl-CoA synthase 11	4.31E-05	**1.10**	1.71E-07	0.91
comp35369_c0	Calcineurin B-like protein	1.20E-02	**1.10**	3.49E-05	**1.26**
comp87642_c0	Aldehyde dehydrogenase family 7 member B4-like	1.64E-16	**1.09**	3.25E-62	**1.13**
comp88862_c0	Pyridoxal biosynthesis protein PDX1	7.77E-15	**1.07**	3.62E-19	0.67
comp88516_c0	Mitochondrial arginine transporter BAC2-like	1.37E-06	0.82	1.49E-30	**1.08**
comp74835_c0	Pyrophosphate-energized inorganic pyrophosphatase			3.41E-02	**1.37**
comp90616_c0	ADIPOR-like receptor CG5315-like	2.20E-03	0.72	2.84E-32	**1.20**
comp75402_c0	Fructose-bisphosphate aldolase			2.29E-65	**1.42**
comp72709_c0	Fructose-bisphosphate aldolase cytoplasmic isozyme			1.78E-06	**1.35**
Oxidative stress related genes					
comp74749_c0	Vacuolar iron transporter homolog	1.81E-24	**5.15**	2.50E-51	**5.29**
comp54647_c0	Peroxidase	5.61E-83	**3.62**	9.01E-137	**3.27**
comp88411_c0	Cationic peroxidase	4.25E-48	**3.26**	4.26E-73	**2.80**
comp44427_c0	MATE efflux family protein FRD3-like	9.69E-58	**2.97**	1.61E-80	**2.60**
comp89738_c0	Heat shock factor protein HSF30	3.42E-19	**2.90**	1.96E-04	**1.18**
comp92757_c0	ATP-dependent Clp protease ATP-binding subunit ClpB homolog 1	1.04E-43	**2.55**	2.80E-174	**2.41**
comp111750_c0	Peroxidase 17-like	1.30E-15	**2.48**	1.22E-48	**3.17**
comp91849_c0	Peroxidase 55	2.99E-47	**2.21**	9.94E-182	**2.62**
comp78537_c0	Calcium-binding protein CML23	3.63E-31	**1.72**	8.73E-144	**2.06**
comp91985_c0	GDP-L-galactose phosphorylase 2	7.26E-31	**1.57**	1.23E-113	**1.49**
comp100177_c0	Peptide methionine sulfoxide reductase B1	7.42E-13	**1.27**	3.05E-59	**1.68**
comp95166_c0	Hsp70-Hsp90 organizing protein 2	9.99E-14	**1.10**	7.41E-55	0.91
comp88862_c0	Pyridoxal biosynthesis protein PDX1	7.77E-15	**1.07**	3.62E-19	0.67
comp80838_c1	Fatty acid dioxygenase AlphaDOX1	1.67E-10	**1.04**	1.67E-04	0.44
comp99015_c0	Full = Selenium-binding protein 1	9.05E-10	**1.04**	4.68E-83	**1.28**
comp101891_c0	GTP-binding protein TypA/BipA homolog isoform X1	2.82E-04	**1.02**	2.77E-19	**1.49**
comp100787_c0	Elongation factor Tu domain 2	4.18E-03	0.97	5.31E-11	**1.45**
comp76346_c0	Catalase	2.36E-08	0.82	4.06E-51	**1.03**
comp73667_c0	Cationic peroxidase 1	2.21E-02	0.72	1.37E-14	**1.17**
comp66299_c0	Catalase			2.08E-05	**1.00**
comp124564_c0	Peroxidase 45			7.97E-04	**1.56**
comp76486_c0	Phospholipid hydroperoxide glutathione peroxidase	5.78E-04	0.63	6.03E-36	**1.08**
comp88505_c0	Full = Inositol-3-phosphate synthase	6.34E-05	0.59	2.82E-78	**1.22**
comp49695_c0	Full = Respiratory burst oxidase homolog protein D			2.01E-04	**1.02**
Proline biosynthetic pathway related genes					
comp32578_c0	Pyrroline-5-carboxylate synthetase (P5CS)	8.50E-06	**1.62**	4.61E-23	**2.39**
comp48238_c0	Pyrroline-5-carboxylate synthetase (P5CS)	3.22E-08	**1.49**	4.95E-50	**2.39**
comp96740_c0	Pyrroline-5-carboxylate synthetase (P5CS)	4.05E-13	0.87	6.40E-123	**1.73**
comp95217_c0	Proline dehydrogenase (ProDH)	1.41E-40	**−2.26**	2.57E-279	**−4.04**
comp76699_c0	Proline dehydrogenase (ProDH)	8.15E-13	**−1.54**	3.61E-75	**−2.82**
comp85493_c0	Proline dehydrogenase (ProDH)	2.14E-04	**−1.17**	2.45E-27	**−2.57**
comp94924_c0	ornithine aminotransferase (OAT)	5.92E-05	0.85	2.30E-30	0.87
comp94345_c0	ornithine aminotransferase (OAT)	8.92E-08	0.86	7.41E-34	0.84
comp100462_c0	pyrroline-5-carboxylate reductase (P5CR)			8.15E-03	0.32
comp93358_c0	pyrroline-5-carboxylate reductase (P5CR)			1.93E-05	0.38

DEGs with |log_2_FC| ≥1 at least at one stage and FDR ≤ 0.05 were included. |Log_2_FC| ≥1 was indicated in bold

Given that prolines are always accumulated as osmolytes in plant to stabilize cell proteins and structures under stresses, we therefore determined the expression changes of key enzyme genes in proline biosynthetic pathway. In our study, unigenes encoding pyrroline-5-carboxylate synthetase (P5CS), ornithine aminotransferase (OAT), pyrroline-5-carboxylate reductase (P5CR) and proline dehydrogenase (ProDH) were identified in gerbera. As shown in Table 5, three *P5CS* genes were induced and their expression level reached to the highest level at stage 4. Both comp32578_c0 and comp48238_c0 showed 1.5–2.4 fold increases of their corresponding expression (log_2_FC values) when compared with the stage 0. *ProDH* genes exhibited an opposite expression level to that of *P5CS*. In addition, expression of OAT and P5CR genes showed only slightly up-regulated during stem bending.

**Fig. 8:**
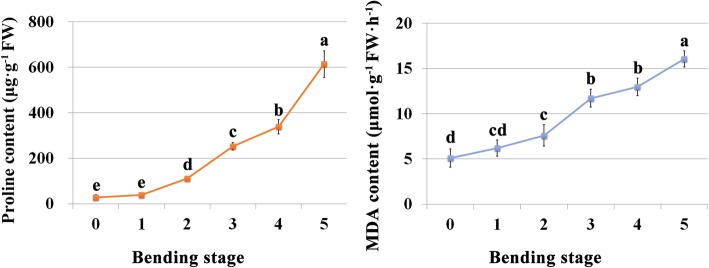
Proline and MDA contents of bent stem segment of gerbera during stem bending. Values are means ± SD (*n* = 5). Lowercase letters indicate significant differences of proline or MDA contents among different bending stages (one-way ANOVA, Duncan’s multiple range test)

To further confirm the accumulation of proline, we measured the content of proline in stem segment (7–12 cm below the floral head) where bending usually occurs in gerbera cut flower during the stem bending. As expected, when compared to the bending stage 0, the proline content increased significantly from bending stage 2. Later in the senescence process it continued to rise markedly (Fig. 8), which was consistent with the transcriptome data mentioned above. We also tested MDA, a toxic molecule and biological marker of oxidative stress. Result showed that its level displayed a rising trend during this process, though its increase was much more moderate than that of proline (Fig. 8). Such results showed that osmotic and oxidative stresses occurred in the bent stem segment during the vase life of gerbera, which may accelerate the bending rate of the stem and speed up the ageing process.

### Expression of genes involved in lignin biosynthesis pathway

We are also interested in important secondary metabolisms which were significantly regulated in stem bending process, especially the lignin biosynthesis pathway. The importance of lignin for stem strength has been reported in several plants, such as rice, rose and gerbera [1, 2, 9, 50]. Monolignols, the source materials for lignin biosynthesis, are synthesized through the phenylpropanoid pathway [51], which was enriched during the stem bending process. In this study, unigenes encoding key enzymes that are involved in biosynthesis of monolignol were identified, such as L-phenylalanine ammonia-lyase (PAL), 4-hydroxycinnamate CoA ligase (4CL), hydroxycinnamoyl CoA shikimate hydroxycinnamoyl transferase (HCT), *p*-coumaroylshikimate 3′-hydroxylase (C3’H), cinnamoyl CoA reductase (CCR), cinnamyl alcohol dehydrogenase (CAD), peroxidase (PER), caffeoyl CoA 3-*O*-methyltransferase (CCoAOMT), and ferulic acid 5-hydroxylase (F5H).

**Fig. 9:**
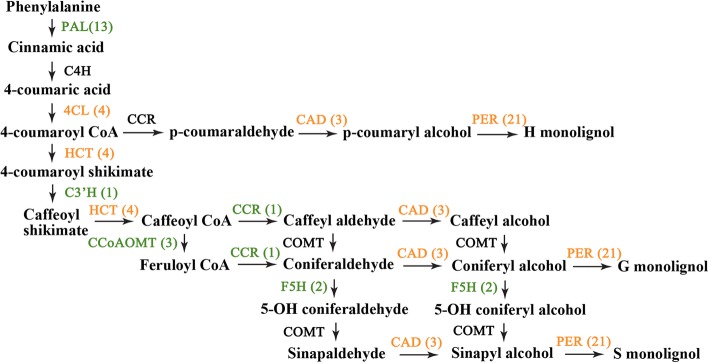
Regulatory changes in monolignol biosynthetic pathway during stem bending process. The enzymes are listed as follows: PAL, C4H (cinnamate 4-hydroxylase), 4CL, HCT, C3’H, CCoAOMT, CCR, CAD, F5H, COMT (caffeic acid 3-O-methyltransferase), PER, and LAC (laccase). DEGs with |log_2_FC| ≥ 1 at least at one stage and FDR ≤ 0.05 are included. Black, genes involved in the pathway but undetectable in our data; orange, some of the genes up-regulated and the others down-regulated; green, down-regulated genes. Numbers in brackets represent numbers of DEGs. The monolignol biosynthetic pathway refers to pathway mentioned by Zhao and Dixon [51]

Thirteen unigenes encoding PAL, the entry enzyme into the phenylpropanoid pathway, were found to be down-regulated during stem bending process (Fig. 9). The unigene comp86307_c0 showed approximately 13-fold decreases of their expression at stage 4 compared to the controls (Additional file 2: Table S3). Two unigenes encoding F5H, the key enzyme responsible for the last hydroxylation of the syringyl-type lignin precursors, were substantially repressed to about 20-fold in expression during the stem bending. CCoAOMT is an important methyltransferase involved in an alternative methylation pathway of lignin biosynthesis. We found that three *CCoAOMT* unigenes were all dramatically down-regulated in this process. Expression pattern of unigenes encoding CCR and C3’H showed the same decreased trend. At the same time, unigenes encoding 4CL, HCT, CAD, and PER displayed inconsistent changes in expression during the stem bending. Such result suggested that the suppression of lignin biosynthesis may exist during this process.

## Discussion

### Illumina sequencing, functional annotation and DEGs analysis

*G. hybrida* is one of the popular cut flowers in the world, and is also the model specie for mechanism investigation of organ growth and development. However, no genomic data is available to date for this species. With the rapid development of RNA-seq technology, transcriptome analysis has become an attractive alternative for in-depth analysis at high resolution. Here, we carried out the RNA-seq analysis of stem transcriptome of *G. hybrida* during the process of stem bending using short-read (Illumina) sequencing. After de novo assembly, we generated 96,492 unigenes with an average length of 662 bp (Table 1). A total of 34,166 (35.4%) unigenes had homologs in at least one of the public databases we searched. It was noticed that the annotations of unigenes of stem in our study were a little less than those of ray floret of gerbera (37,389) [52]. Such result might be due to the difference of genetic information between different organs and development stages of plant, and novel genes or unique pathways which might exist in the stem bending process. In addition, species distribution analysis revealed that the sequences of gerbera stem showed more similarity to *Vitis vinifera* (17.8%) than to other species, which may reflect the evolutionary relationship between *G. hybrida* and *Vitis vinifera* (Fig. 1). Meanwhile, since *Vitis vinifera* (grapevine) is a crop known for a substantial amount of secondary metabolites, its studies on metabolic pathways can be instructive for gerbera as well [53, 54].

Although the global analysis of DEGs during flower organ differentiation [20, 21], flower opening and senescence [55, 56], stress and disease resistance [24, 57] has been performed in many ornamental crops, the data relating to stem bending process in plant remains quite limited. In this study, 9,406 DEGs which differentially expressed at different stem bending stages were identified in *G. hybrida*. It is notable that the number of DEGs progressively increased over the course of stem bending (Fig. 4b), and most DEGs in the comparison of stage 2 vs 0, no matter increased or decreased, were still significantly expressed in the comparison of stage 4 vs 0 (Fig. 4a), indicating a sustaining effect during the whole stem bending process. Furthermore, DEGs (60 up-regulated and 140 down-regulated) identified in common in all the three comparison groups may play essential roles during the stem bending process. In addition, among all the DEGs identified during stem bending process in gerbera, approximately 25.9% of them had no annotated homologs in the aforementioned database we selected (Additional file 4). Some of these genes might be specific to gerbera or represent new stem bending-responsible transcripts which have not been reported in previous studies regarding to other plant species.

### Senescence during the stem bending process

Senescence is a deleterious process, which is efficiently controlled by tightly regulated genetic programs, and is important for the fitness and adaptability of plants [58]. So far, the leaf senescence of plant has been primary focused and well documented [59]. Different from the leaf, stem of gerbera is the supporting organ for the inflorescence, and the typical phenotype of stem senescence of gerbera is stem bending below the floral head. During the stem bending, we found the enrichment of metabolic processes of lipid, protein and nucleic acid (Additional file 5), which generally occur in the senescing leaf and deteriorate the membrane integrity and cellular compartmentalisation at the latter stages of leaf senescence [59]. However, the catabolism of chlorophyll, which is the earliest and most significant cellular degeneration process during leaf aging, was not significantly enriched in the stem bending of gerbera. The possible reason for the difference is that the stem bending of gerbera always occurs before the initiation of stem yellowing. Meanwhile, catabolic process of lignin and pectin, metabolic process of cellulose, as well as modification of cell wall were found enriched during the stem bending (Additional file 5). The enrichment of these pathways suggested that the decrease of the cell wall firmness may cause stem bending in gerbera. In addition, because of the protective mechanism of plant, senescence can occur prematurely when plants are stressed, resulting in decreased yield and quality in plants by limiting the growth phase [60]. The analysis of GO terms during the stem bending also found the enriched pathways related to responses to various types of abiotic stresses, such as osmotic stress and oxidative stress. These environmental cues can accelerate plant senescence partially through regulating the common signalling network involving the signalling molecules, including ETH, ABA, JA and SA [59, 61]. Interestingly, the involvement of ABA in the stem bending was also proved in our work (Fig. 6) and its potential role was discussed in detail in the following paragraph.

At the molecular level, recently, a large number of senescence-associated genes (SAGs) in various plant species are identified [62]. Among these SAGs, numerous TFs, such as NAC, WRKY, MYB, C2H2 type zinc finger, AP2/EREBP [63–67] have been found to exhibit increased expression in senescing leaves, indicating that senescence is governed by highly complex transcriptional regulatory networks. In our studies, all the TF families mentioned above were identified among DEGs. The overlap of the senescence-associated TFs indicated the conservation of the regulatory mechanisms underlying the senescence process across distantly related species. Among the various TFs in our data, NAC and AP2/ERF were the top two largest TF groups, further confirming their importance as global plant senescence regulators. NAC TFs have been implicated in the regulation of various physiological processes including plant defense and senescence [65, 68–71]. Recent evidence in *Arabidopsis* showed that *AtNAP*, a positive NAC regulator of leaf senescence, regulates leaf senescence in part by controlling the expression of *SAG113*. Expression of *AtNAP* is closely associated with the leaf senescence process in *Arabidopsis* [65, 72]. Some AP2/ERF families have been reported to regulate the responses to leaf senescence-associated signaling molecules, including ROS, ETH, JA, ABA, and CK [73, 74]. Taken together, these TFs may lead to fundamental clues about the regulation of senescence, although expression patterns of these TFs were considerable different between different plant species. For example, in cotton *NACs* showed altered expression at various times during senescence [62], whereas in gerbera most *NACs* TFs were up-regulated at the early stage of stem bending and exhibited stable expression at the later stage (Additional file 6). In the *WRKY* family, expression of *AtWRKY70*, a negative regulator of leaf senescence in *Arabidopsis*, was reported to gradually increase and reach a maximum when leaf senescence onset in *Arabidopsis* [75]; whereas transcription level of comp90758_c0 and comp89379_c0, two homologs of *AtWRKY70* in gerbera, didn’t decrease when the stem underwent senescence, suggesting that they may act as positive regulators, rather than negative regulators, of stem senescence in gerbera. It also suggested that different regulatory patterns may exist in stem senescence of gerbera.

### Involvement of ABA in stem bending process

Phytohormones are involved in many different processes throughout the plant life, such as growth, development, senescence and response to environmental stimuli [76]. Various phytohormones, including ETH, ABA, SA, and auxin, have been reported to play important roles in the control of senescence process of plant [77–80]. The transcript level of numerous genes has been found to be altered through the action of phytohormone signaling pathways, as well as by the diverse interactions between phytohormones themselves [81]. In our study, many genes related to hormone pathways, including auxin, CTK, GA, ABA, Eth, BR and SA, showed differential expression during stem bending (Table 4). This result gave a clear picture with regard to the up- or down-regulation of genes associated with various hormones. However, among these hormones, only the majority of DEGs of ABA signal transduction showed a consistently up-regulated trend in expression, suggesting an indispensable role of the ABA in stem bending during postharvest of gerbera cut flower.

ABA is considered as a natural promoter of senescence in many plant species, such as rice, maize and *Arabidopsis* [79, 82, 83]. It has been demonstrated that ABA is strongly tied up to the regulation of developmental senescence in leaf, flower and fruit [72, 84, 85]. As the plant developmentally age, both transcript abundance of genes, which are associated with ABA biosynthesis and signaling [83, 86] and content of endogenous ABA can increase [79]. In *Arabidopsis*, genes encoding the key enzymes in ABA biosynthesis, such as *AtNCED*, *AtAAO1* and *AtAAO3*, were up-regulated during senescence [62]. In our present study, key genes involved in ABA biosynthesis, including *NCED*, *AO*, and *SDR*, also showed significant changes in expression during the stem bending. In addition, expression of the key genes related to ABA signal transduction, including *PP2C* and *SAPK*, were also significantly up-regulated, indicating that the ABA signaling pathway is active during stem bending. It has been reported that function of PP2Cs is complicated in ABA signaling of plants [87]. In *Arabidopsis*, PP2CA was characterized as key negative regulators of ABA signaling, while PP2C-like gene was proved to be a positive regulator [88, 89]. In gerbera, three *PP2Cs* which are up-regulated during stem bending may be senescence-associated *PP2Cs*, which was consistent with previous report on their homologs identified in Iris, an ethylene-independent flower [84]. However, role of these *PP2Cs* in ABA signaling of gerbera is still needed further study. As expected, endogenous ABA level also significantly increased during this process. Thus, we predicted that ABA may act as a positive regulator of stem senescence by increasing the ABA level and up-regulating ABA signaling (Fig. 6; Table 4). On the other hand, stress can induce a senescence response in plants and ABA is one of the principal mediators of the stress responses, such as responses to drought and cold stresses [59]. It has been reported that before senescence, ABA signaling induces multiple stress tolerance processes, including stomatal closure to reduce water loss and suppress senescence; while when the plant ages, ABA signaling changes to induce transcripts, notably *SAG113*, and negatively regulates stress tolerance responses to accelerate senescence [72]. In gerbera, two *SAPK3-like* genes in the ABA signaling were up-regulated during the stem bending, and homolog of *SAPK* in rice has been reported to be involved in stress response in plant [90], suggesting that ABA may mediate the stress induced senescence in gerbera. Given that osmotic stress and oxidative stress were proved to occur in stem bending process, further evidences are still needed to explore role of ABA in interaction between water stress tolerance responses and stem bending in gerbera.

Ethylene plays an important role in regulating flower opening, senescence and abscission in many ethylene-sensitive flowers, such as rose, carnation and orchids. However, researches on role of ethylene in senescence of cut flower of gerbera are few. Earlier report showed that gerbera is an ethylene-insensitive flower [91]. Our previous work also found that effect of exogenous ethylene treatment on the stem bending of gerbera is not significant (data not published). Here, we obtained gene family members encoding ethylene receptors (ETR1, ETR2, EIN4, and ERS1), negative regulator CTR1, positive regulators EIN2, EIN3/EILs (EIN3-like proteins), and the ethylene response factor (ERF); whereas, only expression of *ETR2* and some *ERFs* was found to change significantly during stem bending. The members of *ERFs* showed different expression trends. This result also supported that ethylene might not play an essential role in stem bending process of gerbera.

### Abiotic stresses occurred in stem bending process

It has been reported that one major reason for stem bending of gerbera is adverse water relation in the stem, particularly in the area of bending, which may due to water deficit stress during the postharvest handling, net water loss from stem, and low water uptake in the vase phase [1]. As expected, in our study, expression of some drought tolerance related TF genes, including five DREB/CBF genes and two NF-YA genes, was obviously induced during stem bending process. DREB/CBFs are the most studied TFs involved in drought tolerance and regulates ABA-independent gene expression in response to drought [49]. NF-Y, another group of important regulators coordinating responses to drought stress, is a conserved heterotrimeric complex and consists of NF-YA, NF-YB, and NF-YC subunits [92]. The changes of these TFs were also reported in chrysanthemum under dehydration stress [46]. However, in chrysanthemum, more classic TFs involved in drought stress response, such as *ABA-responsive element binding* (*AREB*) and *abscisic acid-insensitive 5-like* (*ABI5-like*), and *timing of CAB expression 1* (*TOC1*), were found in the dehydration-stressed sample; whereas none of them was detected in our data. Therefore, our result suggested that the stem suffered from a medium water stress, rather than a serious water stress, during the stem bending. Furthermore, our study also proved the occurrence of osmotic stress in the process of stem bending through GO enrichment analysis. Numerous genes involved in osmotic stress response and tolerance are induced (Table 5). It has been reported that the osmotic stress can be imposed on plants due to various abiotic stresses (e.g. drought, high salinity and freezing), and cause damage to growth and development in plant [93, 94]. Though whether the osmotic stress in stem is caused by the adverse water relation is still unclear, the existence of osmotic stress may accelerate the stem bending in gerbera.

Another abiotic stress identified by GO enrichment analysis is oxidative stress. Oxidative stress, which is caused by an imbalance in the generation and removal of reactive oxygen species intermediates (ROIs), is a challenge faced by all aerobic organisms [95]. Sources of ROIs have been identified in plants, including NADPH oxidases, amine oxidases and cell-wall-bound peroxidases, which are tightly regulated and participate in the production of ROIs during processes such as programmed cell death (PCD) and pathogen defense [96]. In oxidative stress gene group in our result, expressions of four peroxidase genes, two cationic peroxidase genes, two catalase genes (CAT) and one glutathione peroxidase gene (GPX) were observed to be up-regulated during stem bending. Both CAT and GPX are important enzymes in the plant antioxidant system which is developed to scavenge the excess superoxide radicals [96], suggesting that the complex antioxidant network and finely tuned ROS accumulation may facilitate appropriate signaling functions, and then promote the stem bending in gerbera. Furthermore, the significant increase of MDA, a toxic molecule and biological marker of oxidative stress, in the bent stem of gerbera also supported this speculation (Fig. 8). In addition, since the oxidative stress may be caused by various abiotic stresses and natural course of senescence in plant [95, 97], further research is needed to explore its formation mechanism during stem bending in gerbera.

Generally, prolines are accumulated and function as osmolytes to stabilize cell proteins and structures in plants under stresses. Proline also acts as a scavenger of free radicals, an energy sink and a stress-related signal [98]. It has been reported that tissue-specific proline synthesis and proline catabolism play a role in promoting growth and maintaining a higher NADP/NADPH ratio at low water potential [99]. In the present study, transcript level of the key enzyme P5CS in the proline biosynthetic pathway showed significant increase during the stem bending process. A marked increase of proline content was also found in the bent stem during stem bending (Fig. 8). The results suggested that proline was needed to maintain cellular redox balance during medium stresses accompanied by stem bending, such as water stress and oxidative stress.

### Comparison of mechanisms behind stem bending in gerbera and heliotropism in other plants

In addition to post-harvested stem bending, plants also have other movements. Tropisms are directed growth-mediated plant movements which contribute to the response to certain environmental stimuli (e.g. light, water and gravity) and ensure the fitness and survival of the plant [100]. Among the tropisms, heliotropism is one of the best-known plant movements [101]. In sunflower, the model plant for studying heliotropism, the apices of elongating vegetative stems shift from east to west during the day and then reorient to face east during the night to follow the Sun’s relative position [100, 102]. Though post-harvested stem bending and heliotropism both belong to the stem movements, mechanisms behind them are quite different.

Firstly, it has been proved that heliotropism is the consequence of differential growth between irradiated and shaded sides of the stem; when growth ceases, the tracking ceases [103]. Thus, stem elongation is necessary for heliotropism. However, the stem bending is not related to elongation in gerbera, because the elongation is confined to the uppermost 10 cm of the stem, which is closer to the head than that of stem bending [1]. And stem elongation occurs earlier than stem bending. Moreover, no effect on stem bending was observed when stem elongation was blocked with SA [1], suggesting that stem bending in gerbera is not a growth-mediated plant movement.

Secondly, different water relations take places in the two movements. For heliotropism, some researchers indicated that one mechanism under this process is the reversible cell-turgor changes [104, 105]. The water reversible gain or loss, which is due to the light-activated reversible ion movements, leads to cell swelling or shrinking, resulting in cell-turgor changes on the east versus the west side of the stem. Such osmotic motor is required by the solar-tracking movement in certain organs, such as the base of leaf petioles (a pulvinus) [105]. For stem bending in gerbera, based on the expression profile of TFs related to water stress tolerance, GO functional enrichment analysis, and determination of proline and MDA, adverse water relation and oxidative stress were proved to be involved in this process, which may be caused by water deficit stress, net water loss from stem, and low water uptake because of xylem blockage by bacteria or material from dead stem cells [1]. The adverse water relation may reduce cell turgor, weaken organ mechanical properties and then accelerate stem bending.

Thirdly, phytohormones participated in these two movements may be different and have different roles. Auxin and gibberellins have been implicated in plant phototropism [106]. The phototropin-triggered lateral transport of auxin from illuminated areas into shaded areas of stem is thought to instigate the heliotropism. This transport causes compensatory growth changes [107] and differential expression of auxin-induced genes, such as *IAA19-*like and *SAUR50-*like genes, on the opposite sides of solar-tracking stems [108–110]. Homologs of these genes are induced by auxin in many plant species [111]. In addition, the *PIN*, *AUX1* and *LIKE-AUX1* (*LAX*) family of genes are also considered to play a role in influx of auxin and the resulting phototropic response through phenotypic identification of mutants. Moreover, the lateral transport of diffusible gibberellin is also greatly induced by unilateral light in the sunflower shoot tip, mediating the differential lateral stem growth [112]. For the stem bending in gerbera, DEGs analysis showed that the auxin signaling seems weaker during stem bending, because most DEGs in auxin signal transduction, including *Aux*/*IAA*s, *GH3*s, and *SAUR*s, were down-regulated in different degree. By contrast, both the up-regulation of DEGs in ABA signaling and the increase of ABA level in our study showed that this process may be regulated mainly by ABA (Table 4, Fig. 6). Moreover, the involvement of ABA in stem bending also supported that the stem bending in gerbera may be the consequence of stem senescence and the adverse water relation in the cut flower. However, more evidences are needed to determine the distribution of ABA in gerbera stem during the stem bending process.

In addition, circadian oscillator is another reason for heliotropism. The circadian clock regulates both auxin levels and auxin signaling partially through controlling expression of auxin-induced genes [113, 114]. Thus, heliotropism, which is the consequence of the interactions between environmental responsiveness and internal circadian oscillator, coordinate physiological processes with predictable changes in the environment to affect plant growth and reproduction [101]. For stem bending in gerbera, another key factor affecting this process is mechanical strength of stem, which mainly depends on wall thickening and the extent of sclerenchyma formation in the stem [1]. Therefore, stem bending of gerbera is caused by a complex set of physiological and cellular events mainly affected by structural characteristics, internal course of senescence, and adverse environmental factors during post-harvest life.

## Conclusions

In this study, we performed large-scale transcriptome sequencing of *G. hybrida* at three successive stages of stem bending using the Illumina sequencing technology. A total of more than 300 million reads were generated and de novo assembled into 96,492 unigenes. A large number of candidate genes involved in stem bending were revealed, including those encoding TFs, important proteins associated with hormone signaling pathway, lignin biosynthesis pathway, and responses to osmotic and oxidative stresses. These findings suggest that stem bending of gerbera plants may be caused by involvement of water stress and regulation of ABA signaling. Collectively, our transcriptome and digital expression profiling of *G. hybrida* provide an important contribution to the current understanding of the molecular regulation of the stem bending and valuable information for functional evaluation of the stem bending-related genes.

## Additional files

Additional file 1: **Figure S1.** Stem-bending stages of *G. hybrida.* **Figure S2.** Length distribution of unigenes in *G. hybrida* transcriptome. (PDF 243 kb)

Additional file 2: **Table S1.** List of primers used in qRT-PCR validation of DEG results. **Table S2.** KEGG mapping of the *G. hybrida* transcriptome. **Table S3.** DEGs related to monolignols biosynthetic pathway during stem bending. (PDF 464 kb)

Additional file 3: Correlation coefficients of two sequencing replicates. (XLS 20 kb)

Additional file 4: List of DEGs between different stem bending stages. (XLSX 2643 kb)

Additional file 5: Significantly enriched GO terms between different stem bending stages. (XLSX 20 kb)

Additional file 6: Up-regulated TFs during stem-bending process. |log_2_FC| ≥ 1 at least at one stage and FDR < 0.05. (XLSX 32 kb)

## Data Availability

All data generated or analysed during this study are included in this article and its supplementary information files. The raw sequence reads were deposited into NCBI SRA database under accession no. PRJNA396423 (https://www.ncbi.nlm.nih.gov/sra/?term=PRJNA396423).

## Abbreviations

4CL: 4-hydroxycinnamate CoA ligase
ABA: Abscisic acid
AO: Aldehyde oxidase
BR: Brassinosteroids
C3’H: p-coumaroylshikimate 3′-hydroxylase
CAD: Cinnamyl alcohol dehydrogenase
CCoAOMT: Caffeoyl CoA 3-O-methyltransferase
CCR: Cinnamoyl CoA reductase
COG: Cluster of Orthologous Groups of proteins
COMT: Caffeic acid 3-O-methyltransferase
CTK: Cytokinins
DEGs: Differentially expressed genes
ETH: Ethylene
F5H: Ferulic acid 5-hydroxylase
FDR: False discovery rate (XLSX)
FPKM: Fragments per kilobase of exon model per million mapped reads
GA: Gibberellin
GO: Gene Onotology database
HCT: Hydroxycinnamoyl CoA shikimate hydroxycinnamoyl transferase
KEGG: Kyoto Encyclopedia of Genes and Genomes
KOBAS: KEGG Orthology Based Annotation System
KOG: Eukaryotic Orthologous Groups
LAC: Laccase
log_2_FC: log_2_ fold change
NCED: 9-*cis*-epoxycarotenoid dioxygenase
NR: Non-redundant protein sequence database
PAL: L-phenylalanine ammonia-lyase
PE: Paired end
PER: Peroxidase
Pfam: Pfam protein families database
qPCR: Quantitative real-time RT-PCR
RNA-Seq: RNA sequencing
SA: Salicylic acid
SDR: Short-chain dehydrogenase/reductase
String: String protein sequence database
SwissProt: Swiss-Prot protein sequence database
TF: Transcription factor
