# Supplementary material for: Transcriptome profiling of Gerbera hybrida reveals that stem bending is caused by water stress and regulation of abscisic acid
Source: BMC Genomics. 2019 Jul 22;20:600. doi: 10.1186/s12864-019-5961-1 (PMC6647082; doi:10.1186/s12864-019-5961-1)
Supplement: Supplementary file 1 — Figure S1. Stem-bending stages of G. hybrida. Figure S2. Length distribution of unigenes in G. hybrida transcriptome. (PDF 243 kb) [file 12864_2019_5961_MOESM1_ESM.pdf]

Additional file 1

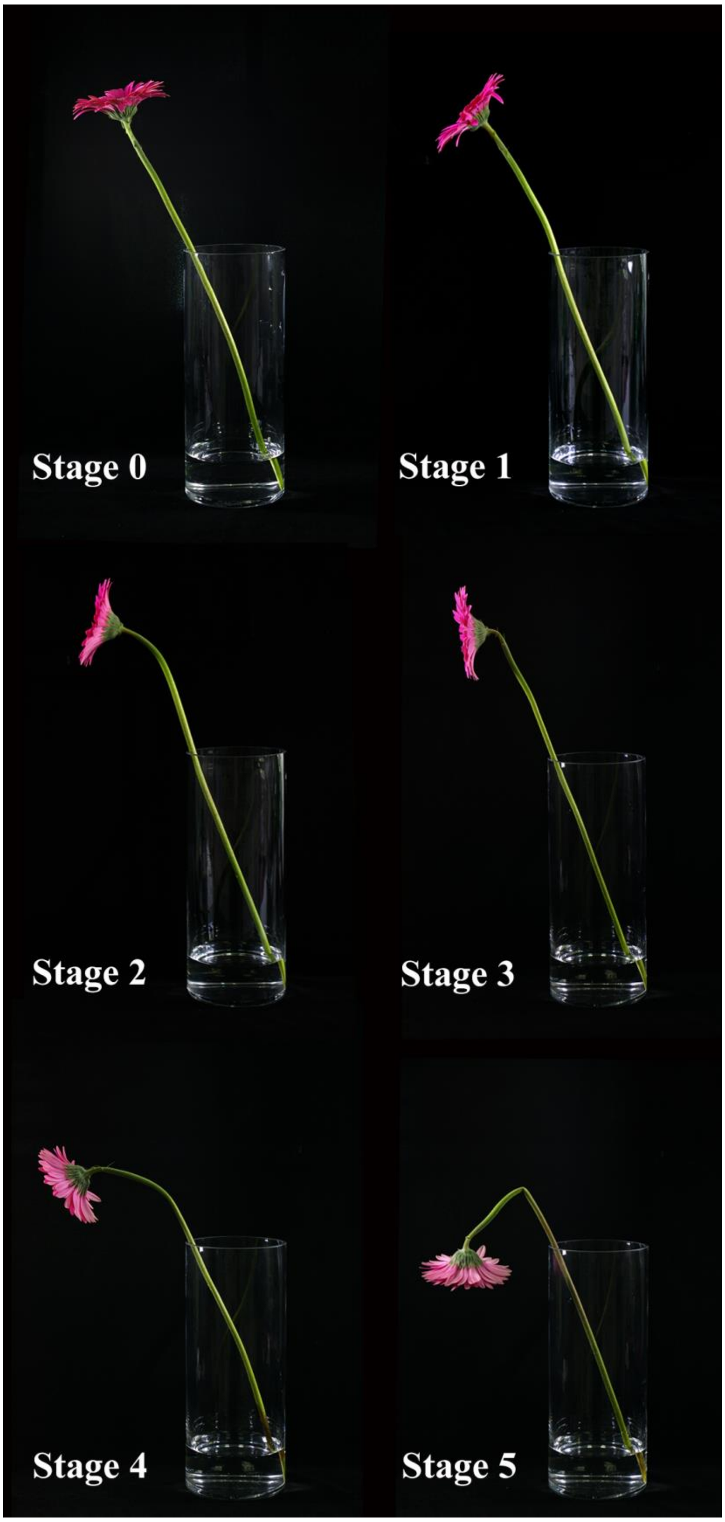

Figure S1 Stem-bending stages of *G. hybrida*. Flowers were individually placed in 20 cm high plastic bottles with the stems standing under an angle of 20 ° with respect to the vertical. The figures showed the stems only beyond the bottle. Stage 0, the angle between floral head surface and horizontal line is less than 30 °; stage 1, the angle is

between 30 ° and 60 °; stage 2, the angle is between 60 ° and 90 °; stage 3, the angle is between 90 ° and 120 °; stage 4, the angle is between120 ° and 150 °; stage 5, the angle is between 150 ° and 180 °

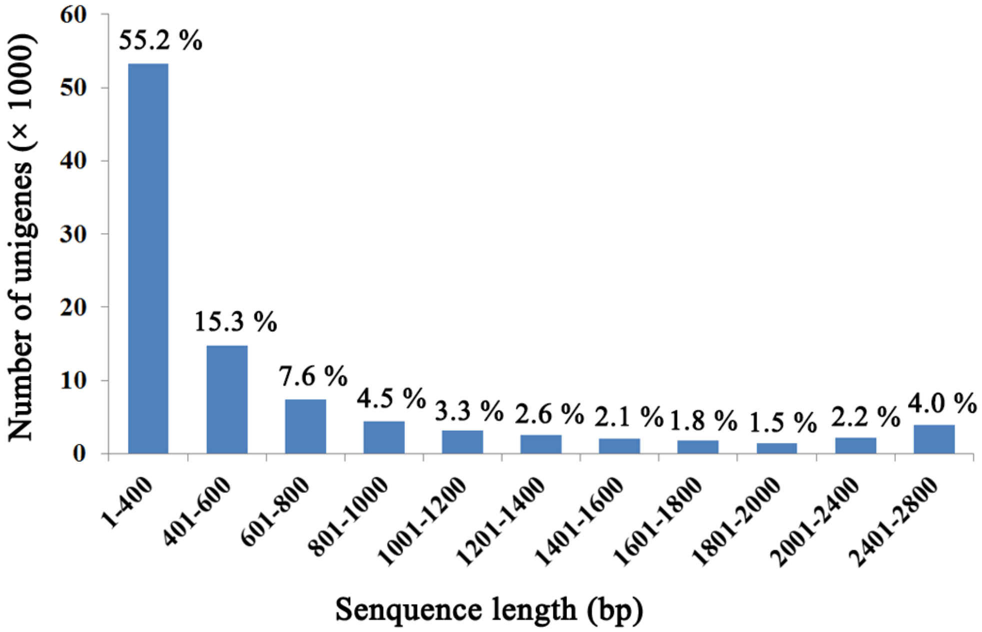

Figure S2 Length distribution of unigenes in *G. hybrida* transcriptome
