# Supplementary material for: Transcriptome profiling of Gerbera hybrida reveals that stem bending is caused by water stress and regulation of abscisic acid
Source: BMC Genomics. 2019 Jul 22;20:600. doi: 10.1186/s12864-019-5961-1 (PMC6647082; doi:10.1186/s12864-019-5961-1)
Supplement: Supplementary file 2 — Table S1. List of primers used in qRT-PCR validation of DEG results. Table S2. KEGG mapping of the G. hybrida transcriptome. Table S3. DEGs related to monolignols biosynthetic pathway during stem bending. (PDF 464 kb) [file 12864_2019_5961_MOESM2_ESM.pdf]

Additional file 2

Table S1 List of primers used in qRT-PCR validation of DEG results

Accession No.	Annotation	Primer set	
		Forward primer (5'-3')	Reverse primer (5'-3')
comp76081_c0	AS2-like protein	TACAAGATGGAACAGGGACTAGATTAT	CCGTCAAAACTGATGGAGTGC
comp84402_c0	LOB domain-containing protein	GACATGGAACTGCGACTGGATT	CACTTAGATCTTCACCGTCAAAACTG
comp80743_c0	ATHB-7	GGTCTTTTCGATCAGTCATGTGG	AGTTAATTACTTAGCAACTCATTACCGTAG
comp32358_c0	ERF15	ACTTCCGACTGTGACAATCTATGG	ATATTCGGGTATTTGTCTATTTTAACTC
comp61667_c0	RAP2-3	GGCACCCCTCTTGCATTTC	GAGATGACTGCTCCACCGC
comp94418_c1	EIN4	TTCTTTTGCAGGGTATTGCTGAT	TGTGGATTACGTTTATAAGCCATTAAC
comp100330_c0	PRUPE	GATTATCATCCAATTCACTCAAAACTC	TCTGATCCGACGGATTTTCCT
comp100389_c0	Feruloyl CoA ortho-hydroxylase 2	GTACGTCAACCTTCCCGACAC	TGAGGGACCCACCAGCAC
comp85722_c0	ARF5	TCAGTTGTTTGAATTTCGAGGGTA	GTGATTTGCGACTGAACATCTTG
comp100009_c0	WEB family protein	GTCACCTTTCTACCAGCTACAGC	CCCTGGCCCCGAGTTTA
comp51383_c0	receptor-like protein kinase	CTCAAACAAGGAGCATGGAGATT	ATTGAAAGAACCGAAGAAGATAAAGA
comp55513_c0	PIP2-7	TCTCGCACTCTATCTTCACCACAG	GGGGTCGTGGTAGTCCTTCG
comp76930_c0	PIP2-1	ACAAGGATAAAGCCTGGGATGA	TCTTAAACTTGAGTGCTGCTTCTG
comp94653_c0	cucumisin	CTTCGCTCCCGTGTCTGG	GATGACTATGTCTAGGTCACATGTCTTAC
comp100475_c0	anion transporter 3	CCAAAGTCTGCTTCATTCACCG	GTTGCCCTTCACTGTTGTATCGT
comp81499_c0	caffeoyl CoA O-methyltransferase	TGGCAGTTGATCCGAGGGT	CCAGTGTGATTTTGCAGTTATGTGT
comp100000_c0	L-ascorbate oxidase homolog	CACAACTCGCCTCACCTCACA	GCGAAGTTTGGCGGCATT
	GhACTIN	AGGAAATCACTGCTCTTGCG	AACAAACTCAACCCTCCAAACC

Table S2 KEGG mapping of the *G. hybrida* transcriptome

NO.	Pathway ID	Pathway	All genes with pathway annotation （10,911）	Percentage
1	ko01100	Metabolic pathways	2,458	22.53%
2	ko01110	Biosynthesis of secondary metabolites	1,192	10.92%
3	ko03010	Ribosome	466	4.27%
4	ko01200	Carbon metabolism	378	3.46%
5	ko01230	Biosynthesis of amino acids	324	2.97%
6	ko04141	Protein processing in endoplasmic reticulum	322	2.95%
7	ko04075	Plant hormone signal transduction	317	2.91%
8	ko00500	Starch and sucrose metabolism	261	2.39%
9	ko03013	RNA transport	242	2.22%
10	ko00190	Oxidative phosphorylation	236	2.16%
11	ko04144	Endocytosis	235	2.15%
12	ko03040	Spliceosome	227	2.08%
13	ko04626	Plant-pathogen interaction	221	2.03%
14	ko00230	Purine metabolism	193	1.77%
15	ko03018	RNA degradation	191	1.75%
16	ko00010	Glycolysis / Gluconeogenesis	190	1.74%
17	ko04110	Cell cycle	181	1.66%
18	ko03015	mRNA surveillance pathway	176	1.61%
19	ko04120	Ubiquitin mediated proteolysis	175	1.60%
20	ko00520	Amino sugar and nucleotide sugar metabolism	162	1.48%
21	ko00564	Glycerophospholipid metabolism	162	1.48%
22	ko00240	Pyrimidine metabolism	150	1.37%
23	ko03008	Ribosome biogenesis in eukaryotes	149	1.37%
24	ko00270	Cysteine and methionine metabolism	148	1.36%
25	ko04145	Phagosome	146	1.34%
26	ko00940	Phenylpropanoid biosynthesis	146	1.34%
27	ko04152	AMPK signaling pathway	141	1.29%
28	ko00620	Pyruvate metabolism	133	1.22%
29	ko00710	Carbon fixation in photosynthetic organisms	130	1.19%
30	ko01212	Fatty acid metabolism	122	1.12%
31	ko04810	Regulation of actin cytoskeleton	121	1.11%
32	ko04024	cAMP signaling pathway	119	1.09%
33	ko00330	Arginine and proline metabolism	113	1.04%
34	ko00360	Phenylalanine metabolism	110	1.01%
35	ko04146	Peroxisome	106	0.97%
36	ko00480	Glutathione metabolism	105	0.96%
37	ko00040	Pentose and glucuronate interconversions	104	0.95%
38	ko00020	Citrate cycle (TCA cycle)	103	0.94%
39	ko04142	Lysosome	102	0.93%
40	ko04014	Ras signaling pathway	101	0.93%

41	ko00260	Glycine, serine and threonine metabolism	98	0.90%
42	ko00562	Inositol phosphate metabolism	98	0.90%
43	ko00561	Glycerolipid metabolism	96	0.88%
44	ko00900	Terpenoid backbone biosynthesis	96	0.88%
45	ko00630	Glyoxylate and dicarboxylate metabolism	95	0.87%
46	ko00052	Galactose metabolism	92	0.84%
47	ko04070	Phosphatidylinositol signaling system	90	0.82%
48	ko00680	Methane metabolism	88	0.81%
49	ko00250	Alanine, aspartate and glutamate metabolism	85	0.78%
50	ko00030	Pentose phosphate pathway	81	0.74%
51	ko03050	Proteasome	81	0.74%
52	ko04010	MAPK signaling pathway	80	0.73%
53	ko00051	Fructose and mannose metabolism	79	0.72%
54	ko03030	DNA replication	76	0.70%
55	ko00565	Ether lipid metabolism	76	0.70%
56	ko00195	Photosynthesis	72	0.66%
57	ko03420	Nucleotide excision repair	71	0.65%
58	ko04712	Circadian rhythm - plant	70	0.64%
59	ko01040	Biosynthesis of unsaturated fatty acids	65	0.60%
60	ko04520	Adherens junction	64	0.59%
61	ko04020	Calcium signaling pathway	64	0.59%
62	ko03440	Homologous recombination	63	0.58%
63	ko03060	Protein export	63	0.58%
64	ko00970	Aminoacyl-tRNA biosynthesis	62	0.57%
65	ko00860	Porphyrin and chlorophyll metabolism	62	0.57%
66	ko00280	Valine, leucine and isoleucine degradation	62	0.57%
67	ko00071	Fatty acid degradation	60	0.55%
68	ko00592	alpha-Linolenic acid metabolism	57	0.52%
69	ko00460	Cyanoamino acid metabolism	56	0.51%
70	ko00130	Ubiquinone and other terpenoid-quinone biosynthesis	56	0.51%
71	ko03320	PPAR signaling pathway	55	0.50%
72	ko04130	SNARE interactions in vesicular transport	55	0.50%
73	ko00310	Lysine degradation	54	0.49%
74	ko00053	Ascorbate and aldarate metabolism	53	0.49%
75	ko00980	Metabolism of xenobiotics by cytochrome P450	53	0.49%
76	ko00380	Tryptophan metabolism	53	0.49%
77	ko00410	beta-Alanine metabolism	52	0.48%
78	ko00061	Fatty acid biosynthesis	52	0.48%
79	ko04540	Gap junction	52	0.48%
80	ko03022	Basal transcription factors	51	0.47%
81	ko03430	Mismatch repair	51	0.47%
82	ko00510	N-Glycan biosynthesis	51	0.47%
83	ko00910	Nitrogen metabolism	50	0.46%

84	ko00400	Phenylalanine, tyrosine and tryptophan biosynthesis	50	0.46%
85	ko02010	ABC transporters	48	0.44%
86	ko00100	Steroid biosynthesis	48	0.44%
87	ko04015	Rap1 signaling pathway	47	0.43%
88	ko04530	Tight junction	47	0.43%
89	ko03020	RNA polymerase	43	0.39%
90	ko00906	Carotenoid biosynthesis	40	0.37%
91	ko00350	Tyrosine metabolism	40	0.37%
92	ko04210	Apoptosis	39	0.36%
93	ko03410	Base excision repair	39	0.36%
94	ko04650	Natural killer cell mediated cytotoxicity	39	0.36%
95	ko00196	Photosynthesis - antenna proteins	39	0.36%
96	ko00600	Sphingolipid metabolism	39	0.36%
97	ko00908	Zeatin biosynthesis	38	0.35%
98	ko00920	Sulfur metabolism	37	0.34%
99	ko00590	Arachidonic acid metabolism	35	0.32%
100	ko02020	Two-component system	35	0.32%
101	ko00941	Flavonoid biosynthesis	34	0.31%
102	ko00513	Various types of N-glycan biosynthesis	34	0.31%
103	ko04140	Regulation of autophagy	33	0.30%
104	ko00904	Diterpenoid biosynthesis	27	0.25%
105	ko00770	Pantothenate and CoA biosynthesis	27	0.25%
106	ko00640	Propanoate metabolism	27	0.25%
107	ko00670	One carbon pool by folate	26	0.24%
108	ko00780	Biotin metabolism	25	0.23%
109	ko04713	Circadian entrainment	25	0.23%
110	ko04978	Mineral absorption	25	0.23%
111	ko00591	Linoleic acid metabolism	24	0.22%
112	ko05020	Prion diseases	24	0.22%
113	ko00450	Selenocompound metabolism	24	0.22%
114	ko00945	Stilbenoid, diarylheptanoid and gingerol biosynthesis	24	0.22%
115	ko00960	Tropane, piperidine and pyridine alkaloid biosynthesis	24	0.22%
116	ko00511	Other glycan degradation	23	0.21%
117	ko00650	Butanoate metabolism	22	0.20%
118	ko04623	Cytosolic DNA-sensing pathway	22	0.20%
119	ko00062	Fatty acid elongation	22	0.20%
120	ko00790	Folate biosynthesis	22	0.20%
121	ko04710	Circadian rhythm	21	0.19%
122	ko00073	Cutin, suberine and wax biosynthesis	21	0.19%
123	ko00340	Histidine metabolism	21	0.19%
124	ko00950	Isoquinoline alkaloid biosynthesis	21	0.19%

125	ko00290	Valine, leucine and isoleucine biosynthesis	21	0.19%
126	ko00909	Sesquiterpenoid and triterpenoid biosynthesis	20	0.18%
127	ko00760	Nicotinate and nicotinamide metabolism	19	0.17%
128	ko01220	Degradation of aromatic compounds	18	0.16%
129	ko00903	Limonene and pinene degradation	18	0.16%
130	ko00730	Thiamine metabolism	18	0.16%
131	ko00563	Glycosylphosphatidylinositol(GPI)-anchor biosynthesis	17	0.16%
132	ko00750	Vitamin B6 metabolism	17	0.16%
133	ko04973	Carbohydrate digestion and absorption	16	0.15%
134	ko00300	Lysine biosynthesis	16	0.15%
135	ko00902	Monoterpenoid biosynthesis	15	0.14%
136	ko00430	Taurine and hypotaurine metabolism	14	0.13%
137	ko00531	Glycosaminoglycan degradation	13	0.12%
138	ko00905	Brassinosteroid biosynthesis	12	0.11%
139	ko04744	Phototransduction	12	0.11%
140	ko00740	Riboflavin metabolism	12	0.11%
141	ko00660	C5-Branched dibasic acid metabolism	11	0.10%
142	ko00072	Synthesis and degradation of ketone bodies	10	0.09%
143	ko00540	Lipopolysaccharide biosynthesis	9	0.08%
144	ko00362	Benzoate degradation	8	0.07%
145	ko00603	Glycosphingolipid biosynthesis - globo series	8	0.07%
146	ko04122	Sulfur relay system	8	0.07%
147	ko03450	Non-homologous end-joining	7	0.06%
148	ko00514	Other types of O-glycan biosynthesis	7	0.06%
149	ko00785	Lipoic acid metabolism	6	0.05%
150	ko00624	Polycyclic aromatic hydrocarbon degradation	6	0.05%
151	ko00140	Steroid hormone biosynthesis	6	0.05%
152	ko00523	Polyketide sugar unit biosynthesis	5	0.05%
153	ko04977	Vitamin digestion and absorption	5	0.05%
154	ko00942	Anthocyanin biosynthesis	4	0.04%
155	ko00471	D-Glutamine and D-glutamate metabolism	4	0.04%
156	ko00966	Glucosinolate biosynthesis	4	0.04%
157	ko00604	Glycosphingolipid biosynthesis - ganglio series	4	0.04%
158	ko00253	Tetracycline biosynthesis	4	0.04%
159	ko00232	Caffeine metabolism	3	0.03%
160	ko00361	Chlorocyclohexane and chlorobenzene degradation	3	0.03%
161	ko00944	Flavone and flavonol biosynthesis	3	0.03%
162	ko00965	Betalain biosynthesis	2	0.02%
163	ko00534	Glycosaminoglycan biosynthesis - heparan sulfate / heparin	1	0.01%
164	ko00943	Isoflavonoid biosynthesis	1	0.01%

Table S3 DEGs related to monolignols biosynthetic pathway during stem bending

Putative function	Gene ID	Expression value (fpkm)			stage 2 vs stage 0		stage 4 vs stage 0		Regulation
		stage 0	stage 2	stage 4	FDR	log_2 FC	FDR	log_2 FC	
PAL	comp81171_c0	111.33	24.84	9.29	4.79E-42	-2.94	1.30E-282	-3.75	Down
	comp57516_c0	47.41	14.93	6.47	1.90E-16	-1.65	2.67E-114	-3.05	Down
	comp82886_c0	15.13	6.92	4.65	3.78E-05	-1.03	1.11E-26	-1.87	Down
	comp86307_c0	108.15	16.42	7.93	2.47E-68	-3.35	0.00E+00	-3.95	Down
	comp105907_c0	13.62	2.24	2.23	1.22E-06	-1.83	6.65E-14	-2.86	Down
	comp95435_c0	315.84	152.28	71.26	1.67E-20	-1.23	2.69E-116	-2.37	Down
	comp71426_c0	9.95	5.40	3.06	2.03E-02	-1.32	1.23E-08	-1.91	Down
	comp91289_c0	227.28	114.68	62.84	4.86E-11	-1.01	5.15E-53	-2.05	Down
	comp98450_c0	893.23	461.68	263.66	3.50E-18	-1.06	1.30E-81	-1.83	Down
	comp54105_c0	11.22	9.67	3.87	2.47E-01	-0.82	9.35E-09	-1.77	Down
	comp51743_c0	9.03	2.90	2.35	1.04E-01	-0.87	5.56E-05	-2.17	Down
	comp47912_c0	4.18	0.85	0.50	3.80E-01	-1.49	4.51E-03	-3.23	Down
	comp32864_c0	55.32	30.04	23.96	2.35E-03	-0.79	6.27E-16	-1.43	Down
4CL	comp67287_c0	2.82	9.68	7.35	5.93E-07	1.18	8.36E-11	1.12	Up
	comp67784_c0	171.51	36.22	21.89	1.65E-38	-2.11	2.25E-274	-3.15	Down
	comp84378_c0	228.47	57.97	28.26	3.89E-44	-2.02	0.00E+00	-3.19	Down
	comp113390_c0	3.65	1.31	0.17	7.31E-01	-0.69	2.94E-05	-4.23	Down
CCR	comp80364_c0	396.09	150.60	144.19	2.94E-30	-1.48	5.51E-163	-1.64	Down
HCT	comp81953_c0	8.69	26.59	31.11	1.19E-11	1.59	4.29E-30	1.65	Up
	comp91808_c0	8.06	36.76	31.84	4.73E-27	1.93	7.37E-54	1.80	Up
	comp91548_c0	28.14	101.14	134.15	4.40E-24	1.82	8.60E-158	2.06	Up
	comp32443_c0	302.49	216.19	144.67	3.27E-10	-0.73	5.02E-109	-1.24	Down
C3'H	comp94602_c0	264.78	91.99	74.04	2.47E-46	-1.73	1.67E-254	-2.02	Down
CAD	comp83255_c0	3.39	12.87	4.50	1.40E-08	1.72	9.23E-01	0.23	Up
	comp78483_c0	11.87	25.57	26.44	9.98E-09	1.12	2.74E-28	1.19	Up
	comp98744_c0	620.98	164.95	112.47	5.11E-56	-2.02	1.85E-284	-2.64	Down
CCoAOMT	comp81499_c0	206.20	18.90	13.56	1.76E-70	-3.45	0.00E+00	-4.11	Down
	comp91683_c0	1012.59	145.24	92.10	8.35E-128	-3.10	0.00E+00	-3.60	Down
	comp92787_c0	1924.60	523.37	316.20	9.95E-65	-1.95	1.36E-306	-2.59	Down
F5H	comp69171_c0	12.08	0.43	0.67	1.74E-38	-4.02	4.23E-100	-4.33	Down
	comp92126_c0	396.08	39.42	20.81	4.27E-147	-3.47	0.00E+00	-4.43	Down
PER	comp54722_c0	1.13	14.64	12.99	2.78E-11	3.86	1.07E-11	3.27	Up
	comp54647_c0	2.72	40.18	29.78	5.61E-83	3.62	9.01E-137	3.27	Up
	comp88411_c0	2.93	28.23	23.04	4.25E-48	3.26	4.26E-73	2.80	Up
	comp111750_c0	0.90	6.45	9.27	1.30E-15	2.48	1.22E-48	3.17	Up
	comp91849_c0	12.33	66.52	85.80	2.99E-47	2.21	9.94E-182	2.62	Up
	comp73667_c0	7.52	13.58	19.27	2.21E-02	0.72	1.37E-14	1.17	Up
	comp84347_c0	1072.11	278.75	276.39	6.65E-62	-2.08	2.05E-293	-2.14	Down
	comp125492_c0	6.79	0.42	0.81	8.91E-15	-3.21	1.67E-25	-3.24	Down
	comp76181_c0	10.32	1.87	2.35	3.18E-16	-2.50	7.76E-40	-2.31	Down

comp86970_c0	129.84	64.12	69.80	5.12E-18	-1.15	1.56E-58	-1.08	Down
comp87613_c0	7.03	0.36	0.22	4.07E-24	-3.51	6.08E-62	-5.16	Down
comp88587_c0	116.75	24.95	19.06	4.92E-59	-2.36	0.00E+00	-2.79	Down
comp31721_c0	4.58	0.28	0.07	8.38E-06	-3.20	3.46E-15	-5.78	Down
comp78747_c0	426.12	3.07	0.84	4.42E-203	-8.53	0.00E+00	-9.14	Down
comp376727_c0	0.71	0.06	0.42	3.79E-02	-2.80	2.79E-01	-0.98	Down
comp105366_c0	8.20	5.17	11.46	3.95E-04	-1.02	7.37E-02	0.30	Down
comp34032_c0	19.84	0.15	0.19	2.83E-50	-6.21	1.02E-151	-6.81	Down
comp91512_c0	73.28	19.22	23.38	2.21E-34	-1.97	5.95E-46	-1.81	Down
comp90192_c0	20.52	0.47	0.78	1.15E-49	-4.66	2.85E-140	-4.92	Down
comp103037_c0	55.14	2.15	3.56	1.70E-73	-4.45	3.80E-257	-4.12	Down
comp84212_c0	28.13	20.41	15.85	3.36E-04	-0.64	1.68E-26	-1.02	Down

DEGs with $|\log_2 FC| \geqslant 1$ at least at one stage and FDR ≤ 0.05 were included
